# Zebrafish *mecp2* null-mutation increases anxiety and cortisol levels but no change in adult social preference and larval chemically-induced hyperlocomotion

**DOI:** 10.1186/s12868-025-00946-8

**Published:** 2025-07-01

**Authors:** Soaleha Shams, Pierre Cronell, Jenny Landin, Thomas Pietri, Adrian Ekehorn Gimdal, Petronella Kettunen, Lars Westberg

**Affiliations:** 1 Department of Pharmacology, Institute of Neuroscience and Physiology, Sahlgrenska Academy, University of Gothenburg, Box 431, 405 30 Gothenburg, Sweden; 2 Department of Biochemistry & Molecular Biology, Mayo Clinic, Rochester, MN USA; 3 Elsevier B.V, Radarweg 29a, 1043 NX Amsterdam, The Netherlands; 4 Department of Psychiatry and Neurochemistry, Institute of Neuroscience and Physiology, Sahlgrenska Academy, University of Gothenburg, Gothenburg, Sweden; 5 Department of Neuropsychiatry, Sahlgrenska University Hospital, Region Västra Götaland, Gothenburg, Sweden

**Keywords:** Rett syndrome model, Social behaviour, Locomotion, Anxiety, Pentylenetetrazol, Seizure, Cortisol

## Abstract

**Background:**

Methyl CpG binding protein 2 (MECP2) is an essential global modulator of transcription and mutations in *MECP2* are the most common cause of Rett syndrome, an X-linked neurodevelopmental disorder. Patients diagnosed with Rett syndrome have increased risk for epilepsy as well as problems with anxiety and social communication. Using the zebrafish *mecp2*^*Q63X*^ line, this study aimed to increase our understanding of the role of Mecp2 function in regulation of pharmacologically-induced hyperlocomotion, developmental social preference, and adult socialization, anxiety-related behaviour, and baseline cortisol levels. To determine responses of *mecp2*^−/−^ zebrafish to a stimulating convulsant, general locomotor activity was measured at 5 days post-fertilization (dpf) in sibling *mecp2*^+/+^, *mecp2*^+/−^, and *mecp2*^−/−^ fish after treatment with a GABA_A_ receptor antagonist pentylenetetrazol (PTZ) at varying concentrations. Responses to social stimulus were investigated in juvenile (21 dpf) and adult *mecp2*^−/−^ and *mecp2*^+/+^ fish. Anxiety responses to a novel tank and whole-body cortisol levels were also measured in adult *mecp2*^−/−^ and control *mecp2*^+/+^ zebrafish.

**Results:**

The behavioural tests showed that *mecp2*^−/−^ zebrafish displayed hypolocomotion at the larval stage, along with increased freezing time and thigmotaxis, and higher whole-body cortisol levels in adulthood. However, the hyper-locomotion response to PTZ at 5 dpf and social preference for visual social stimulus at 21 dpf and in adulthood were not affected by the lack of functional Mecp2.

**Conclusions:**

Functional Mecp2 modulated larval locomotion and behavioural anxiety at different ages and adult cortisol levels, but *mecp2* null-mutation did not alter adult locomotion and socialization, and developmental sociability and PTZ-induced hyperlocomotion in zebrafish. Given the variability reported in patients and in rodent *Mecp2* knockout models, studies using zebrafish can explore vital elements of MECP2’s role across development and improve our understanding of neural mechanisms underlying neurodevelopmental disorders.

## Introduction

MECP2 (methyl CpG binding protein 2) is essential for maturation and optimal functioning of neurons and non-neuronal cells as a global transcription regulator [1, 2]. Under- and overexpression of MECP2 is linked to Rett Syndrome, MECP2 Duplication syndrome, and various other neuropathologies and developmental disorders [3–5]. The X-linked *MECP2* gene is ubiquitously expressed, and the MECP2 protein binds to methylated CpG dinucleotides and other non-CpG motifs [1], and modulates activity-dependent neuronal transcription of genes [2] in the vicinity of the methylated sequence. Upon synaptic activity, MECP2 gets phosphorylated which controls its repressor activity through recruitment of histone deacetylase and the corepressor SIN3A, and in turn regulates experience-dependent dendritic growth and spine maturation in the brain [6–8]. Over 600 mutations in the *MECP2* gene have been identified as the major cause [5] of the progressive neurodevelopmental disorder Rett syndrome [3, 4].

In humans, Rett syndrome is a monogenic disorder that primarily affects girls as the males fetuses do not tend to survive [9]. Following seemingly typical prenatal and infant development, Rett syndrome has an early onset [~ 6–12 months of age] and a characteristic disease pattern of rapid and progressive developmental delay, regression, and loss of motor and language skills [between 12–30 months] along with gait impairments and repetitive hand stereotypies [10, 11]. This regression is followed by a longer plateau stage with stable abilities and symptoms for several years and can be followed by a later deterioration stage [5–25 years of age] with further reduction in motor skills [10–12]. Although the first disease-modifying treatment Trofinetide, a synthetic analog of glycine–proline–glutamate, the N-terminal tripeptide of the insulin-like growth factor 1 protein [IGF1], was approved by the FDA recently [13, 14], treatment of Rett syndrome have been mainly symptomatic [15].

The typical symptoms of this syndrome are lack of intentional hand movements, intellectual disability, language difficulties, repetitive movements, autonomic dysfunction, poor eye contact, and social behavioural problems [16–18]. Additionally, seizures, heart problems, gastrointestinal and sleep abnormalities, heightened anxiety and stress responses [19, 20], and aberration in stress hormone, cortisol [21–23], have been reported in these patients. While abrupt changes in mood, irritability, and heightened anxiety are not clinically diagnostic for Rett syndrome, recent natural history and care-giving studies have identified these to be significant concerns by care-givers and commonly treated using anxiolytics and anti-depressants [10–12, 19, 20, 24]. Therefore, there is a great need for model systems for evaluating pharmaceutical, biologic, and gene therapies that may relieve and ameliorate symptoms of Rett syndrome, including motor, social, mood, and anxiety symptoms.

Animal models that mimic the phenotype of Rett syndrome have been generated by knocking out *Mecp2* in mice [25–30], rats [31, 32], and primates [33]. In addition, disease models using human neurons derived from stem cells (iPSCs) from patients with Rett syndrome offer the potential of high-throughput drug screening [34–38] while patient-derived, gene-edited, and knock-out cortical organoid models can also allow better recapitulation of variability and complexity seen in Rett syndrome [39–42]. Extensive animal studies mainly in mice reveal that loss of *Mecp2* expression results in cognitive impairments [43, 44], abnormal gait and reduced locomotor activity [45], increase vulnerability to stress and premature death [2, 46, 47], and alters specific aspects of synaptic physiology and neurotransmission that may impact the balance of excitation/inhibition [E/I balance] in the brain [48–50] but specific signalling pathways underlining behavioural and neuronal abnormalities phenocopied in mutant mice remain unknown. Additionally, similar to many other neuropathologies, promising treatments from various mice studies focusing on *Mecp2* show no or limited translational effectiveness. The lack of phenotypic complexity in *in-vitro* cell and coculture models and the variance, subtlety, and mildness in phenotypes [51, 52] of onset, progression, and lethality reported in hemizygous male and heterozygous female mouse models necessitate additional model systems with sufficient phenotypic complexity and practical simplicity.

The zebrafish offers a complementing model with several advantages over mammalian and cell/culture model systems as an excellent tool with availability of sophisticated genetic manipulation techniques, high-throughput screening approaches [53, 54], and detailed investigations of behaviours, such as locomotion and sociability [55–60], and anxiety [45, 61, 62]. Zebrafish Mecp2 shows similarity [63] in amino acid sequence [43%] and expression patterns in adult brains and across development, and the *mecp2* gene is autosomal [chromosome 8 and not X-chromosome] [64]. Null *mecp2*^−/−^ zebrafish are viable and fertile allowing both sexes to be studied, along with feasibility of studying potential epigenetic and transgenerational effects.

To study neurodevelopmental roles and to identify previously unknown functions and novel mechanisms of MECP2, unique features of the zebrafish can be particularly important such as producing large numbers of externally fertilized transparent embryos that can be tracked experimentally into juvenile and adult stages. The small size, rapid external development, and robust locomotor, anxiety, and feeding behaviours exhibited by larval zebrafish within a few days post-fertilization (dpf) could be greatly revealing in studying the earliest effects of MECP2-deficiency that eventually leads to pathology. Additionally, while juvenile and adult zebrafish show a wide range of complex social, affective, and cognitive behaviours, the adult zebrafish brain retains higher level of neurogenesis mimicking mammalian embryonic brain [65]. Zebrafish are also diurnal, rely on their vision more than olfaction for social perception and processing, and have metabolic, physiological, and epigenetic mechanisms that more closely mirror human circadian oscillation. Such advantages may be leveraged to provide greater understanding of variation in phenotypes seen in affected patients, differentiate between conflicting results seen in mouse models, and, shed more light on disparities reported between patients and mouse and other mammalian models.

Previous work with zebrafish *mecp2*-null mutants have showed recapitulation of milder phenotypes of human Rett syndrome symptoms. Studies of zebrafish Mecp2 including development of key mutant and morpholino-mediated models, environmental factors and pleiotropic interactions, and relevant upstream regulators of *mecp2* and affected downstream pathways are summarized in the timeline in Table 1. Aside from confirmation of motor impairments and changes in anxiety behaviour in the ENU-derived and TILLING-selected *mecp2*-null zebrafish *mecp2*^*Q63X*^ model [64], visual function and energy metabolism have also been suggested to be impaired [66, 67]. In the current paper, to identify current progress, common themes, and any gaps and to gain a more comprehensive understanding of existing literature, particularly the *mecp2*^*Q63X*^ model, we first briefly reviewed all published zebrafish Mecp2 studies (Table 1). To further substantiate the use of *mecp2*^−/−^ zebrafish as a model for Rett syndrome and to investigate basic visual function, we measured general locomotion and anxiety-related behaviours at three different ages [5 dpf, 21 dpf, and 6-months of age], PTZ-induced hyperlocomotion in larval 5 dpf fish, social preference for vision-based social stimuli when zebrafish start shoaling [21 dpf] and social, anxiety, and locomotion behaviour and cortisol levels in adulthood in *mecp2*^−/−^ zebrafish.

**Table 1:** Chronological history of key developments and significant discoveries of zebrafish Mecp2 structure, expression, and function

Publication, Year	Summary of zebrafish Mecp2 findings
Hendrich & Tweedie, 2003 [68]	Conducted phylogenetic analysis of genomic DNA methylation, methyl-CpG-binding domains [MBDs], and MBD proteins in invertebrate and vertebrates and confirmed presence of zebrafish Mecp2.
Cloverdale et al., 2004 [63]	Showed 43.7% similarity in amino acid [AA] sequences [85.9% in the critical MBD] and similar brain expression patterns between zebrafish Mecp2 and mammalian MECP2α isoform by *in-situ* hybridization in embryonic and adult zebrafish brains. Confirmed *mecp2* expression from 24–72 hpf and substantially higher in adult brains.
**Pietri et al., 2013** [64]	Established conserved synteny and the first zebrafish *mecp2*-null model [ENU-mutagenesis and TILLING-selected *mecp2*^*Q63X*^] with a C187T mutation that truncates the protein at AA 63. Reported locomotion anomalies in 24 and 51 hpf embryos and 6 dpf larvae [reduction in activity, velocity, and thigmotaxis] in the *mecp2*^*Q63X*^ larvae. Also suggested altered Excitation/Inhibition balance and shorter lifespans in *mecp2*^−/−^ fish.
Pappalardo-Carter et al., 2013 [69]	Reported that ethanol exposure during gastrulation did not affect *mecp2* mRNA level at 24 and 48 hpf.
Yang et al., 2013 [70]	Transcriptomic Analysis compared wildtype zebrafish embryos from cleavage (64/128-cell) to 7 dpf. Found *mecp2* expression is inactivated in early gastrulation, then reactivated during late embryonic stages, indicating link to critical time-sensitive functions during neurological development.
Huang et al., 2013 [71]	Conducted a large-scale screen focusing on zebrafish orthologues of human chromatin regulators of progenitor stem cells and differentiation during hematopoiesis in zebrafish embryos. Employing *mecp2* exon-2 morpholinos, linked zebrafish Mecp2 to orthologues protein–protein interactions in human BAF-PBAF and HDAC-NuRD complexes.
Gao et al., 2015 [72]	Morpholino-mediated knockdown and overexpression in wildtype zebrafish and F0-CRISPR-mediated S143-mutation linked Mecp2 to increased neuronal differentiation and astrogenesis, reduced neurogenesis, suppression of downstream Id1-Her2 axis and upregulation of Notch signaling during development. Reported co-localization of immunolabeled Mecp2 with HuC [marker for mature neurons] in adult zebrafish brain by creating an antibody [IHC & WB] that recognizes an epitope [AA 170–223] of zebrafish Mecp2.
**Leong et al., 2015** [73]	Mecp2 deficiency caused by null mutation [*mecp2*^*Q63X*^] and morpholino-mediated silencing led to defects in peripheral branching of the trigeminal sensory neurons at 24 hpf and delayed sensory responses to tactile stimuli at 48 hpf. Chromatin immunoprecipitation [ChIP] assays with gene promotors confirmed that these sensory defects were mediated by axon growth regulators [Sema5b and Robo2].
Laing et al., 2016 [74]	Chronic bisphenol-A exposure [BPA, 15 days at 0.1 mg/l] of adult fish led to reduction in global DNA methylation in gonads and change in transcription of *mecp2* in male zebrafish livers [but not in female livers].
Wang et al., 2016 [75]	Maternal exposure to water-soluble fraction of crude oil [5 μg/l] and lead [20 μg/l] for seven days before fertilization resulted in down-regulation of whole-body *mecp2* mRNA expression in 15 dpf larval zebrafish, along with increases in anxiety and locomotion.
Jimenez-Gonzalez et al., 2016 [76]	Studied negative regulation of zebrafish *mecp2* by post-transcriptional regulator microRNAs, and effect of inhibition of *mecp2* on Bdnf in morphine-exposed embryos [10 nM between 5–48 hpf]. Reported binding of miRNAs-212/132 to zebrafish *mecp2* exon-3 fragment using luciferase assays in HEK293 cells and proposed degradation of *mecp2* mRNA led to higher levels of Bdnf and its receptor, TrkB, in morphine-treated embryos.
Garcia-Concejo et al., 2016 [77]	In contrast to their earlier work above [Jimenez-Gonzalez et al., 2016], suggested that Mecp2 mediates morphine-induced regulation of mμ opioid receptor [Oprm1] and microRNA-212/132 after morphine-exposure in zebrafish embryos [5–24 hpf and 5–48 hpf at 10 nM or 10 uM].
**Cortelazzo et al., 2017** [66]	Explored pleiotropic roles of human MECP2 using proteomic analysis of *mecp2*-null mutation [*mecp2*^*Q63X*^] in 8 dpf and adult zebrafish and showed changes in expression of proteins involved in energy metabolism, redox balance, and structure and function of cardiac and skeletal muscle, pathways relevant for motor impairments in affected Rett syndrome patients and in line with reports from mouse models studies. Proposed potential visual impairment due to reduction in Cryba2, which maintains transparency of vertebrate lens, in *mecp2*^*Q63X*^ larval and adult zebrafish.
Nozawa et al., 2017 [78]	Using morpholino-mediated knockdown, reported decreased motor activity, reduced and aberrant axon branching in *mecp2* morphants, compared to uninjected controls. Zebrafish *mecp2* gene was ubiquitously expressed and affected axonal growth of sensory and motor [primary and secondary] neurons and synapse formation at 28 and 72 hpf via regulation of *bdnf* gene expression. Injections of human *MECP2* mRNA and knocking down *bdnf* could rescue some of the synaptic and axonal abnormalities.
**Van der Vaart et al., 2017** [79]	Combining null mutants [*mecp2*^*Q63X*^] and fluorescently labeled microglia and macrophages, established zebrafish Mecp2 as an immunological regulator by downregulation of *tnfa* [but not *il1b* or *il10*], even with an immune stimulation during development [6 hpf—7 dpf]. Dysregulated pro- and anti-inflammatory cytokines, bile-related GI tract discoloration, and differences in body length [only at 2dpf], inflammation marker *crp*, and number of total and GI tract neutrophils were also reported [4-7dpf]. Transcriptomics analysis of *mecp2*^−/−^ and wildtype controls [at 6 hpf] revealed *mecp2*-deficiency led to differential expression of 8000 + genes that link to a large range of biological processes.
Laing et al., 2018 [80]	Reported sex-differences in transcript level of *mecp2* [along with other epigenetic regulators including *dnmt1*, *dnmt3*, *mbd2*, *mbd3a*, and *hdac1*] in wildtype adult zebrafish gonads but not livers; sevenfold overexpression of *mecp2* in ovaries compared to testes.
Sakai et al., 2018 [81]	Review article examined studies with functional analysis of genes linked to autism, epilepsy, and intellectual disability using embryonic and larval zebrafish, with critical comparisons of *mecp2* morphants and mutants.
Pisera-Fuster et al., 2020 [82]	Modeling human smoking behaviour with conditioned place preference [CPP] in wildtype zebrafish, these researchers reported that continuous and intermittent [for 14 days, 30 uM] exposure to nicotine led to an eightfold and fivefold increase, respectively, in levels of *mecp2* mRNA in dopaminergic mesolimbic reward pathways in the adult zebrafish brains. Treatment with histone acetylase inhibitor phenylbutyrate [PhB] reduced *mecp2* and *hdac1* mRNA levels, suggesting link between zebrafish *mecp2* and *hdac1*.
Ross et al., 2021 [83]	Examined DNA methylomes and transcriptomes of larval and adult zebrafish and showed via RNA-seq analysis that brain *mecp2* transcript levels increased from 3 weeks to adulthood, corresponding with increases in non-CpG methylation and neuronal differentiation, and comparable to mammalian expression patterns.
Baronio et al., 2022 [84]	Studied function of zebrafish monoamine oxidase [mao, enzyme that metabolizes dopamine, serotonin, norepinephrine, and histamine] and reported that *mecp2* mRNA levels from RT-qPCR analysis of adult brains were similar for wildtype *mao*^+/+^ and heterozygous *mao*^+/−^ siblings.
Gonçalves et al., 2022 [85]	Compared various wildtype zebrafish strains [AB, TU, WIK, LEO, TL, 5D] to examine single nucleotides polymorphisms associated with social and non-social behaviours but findings attributed to *mecp2* SNPs are mislabeled [rs180034095, rs180034118, rs180034123 are located on chromosome 16, intronic variants in dopamine receptor D2-like, *drd2l* gene].
Gabellini et al., 2022 [86]	Generated a zebrafish *setd5* mutant line to study human haploinsufficiency of SETD5, a potential histone-modifying enzyme linked to autism and intellectual disability, and reported no differences in *mecp2* mRNA levels from RT-qPCR analysis between wildtype *setd5* ^+/+^ and heterozygous *setd5*^+/−^ adult zebrafish whole-brains.
Varela et al., 2022 [87]	Relative gene expression of *mecp2* was upregulated in heterozygous and homozygous *cdkl5* mutants, compared to wildtype adult fish. *Cdkl5* [*cyclin dependent kinase like 5*] mediates Mecp2 phosphorylation and Mecp2 can repress *cdkl5* transcription.
Adrião et al., 2023 [88]	Created single and double loss-of-function *mef2c* mutant and fluorescent reporter zebrafish lines to study human MEF2C haploinsufficiency syndrome [affected patients have decreased expression of *MECP2* and *CDKL5*]. qPCR analysis showed decreased *mecp2* and *cdkl5* gene expression in all *mef2c* mutants at 3 dpf.
Pramanik et al., 2024 [89]	Review article highlighting use of zebrafish for better understanding of pathophysiology, disease modeling and effective therapies for Rett Syndrome and comparison of zebrafish findings with mouse models studies.
**Santistevan et al., 2024** [67]	Employing transcriptomic and behavioural assays of 5–7 dpf [*mecp2*^*Q63X*^] mutants, concluded Mecp2 to be essential for thigmotaxis [but not general locomotion, startle response or sensory filtering] and emphasized link to responses to visual stimuli.
**Privat et al., 2024** [90]	Studied optic tectum and prey-capture behaviour of [*mecp2*^*Q63X*^] mutants and reported reduced functional connectivity in the optic tectum neural circuits, impairments in visual response to prey and stimulus discrimination, and more mistakes during capture in 5–7 dpf fish.
**Shams et al., 2025** Current Paper	Utilizing [*mecp2*^*Q63X*^] null-mutants, we studied PTZ-induced locomotion in 5 dpf larvae, determined social preference in 21 dpf larvae, and tested adult behavioural responses to the open-field, to social stimuli, and to a novel tank. Our results show *mecp2*^*−/−*^ larvae were hypoactive but not differentially affected by PTZ-exposure. Ontogeny of social behaviour at 21 dpf and expression of social behaviour in adulthood was not affected in mutants either but they showed more anxiety-related behaviours.

Review and summary of existing literature with development of mutants and morpholinos, genotoxic and environmental influencers, and established links with upstream regulators and downstream affected pathways are stated. Publications using zebrafish with the same *mecp2*-null mutation [*mecp2*^*Q63X*^] as the current study are highlighted in bold font. [hpf = hours post fertilization, dpf = days post fertilization]

## Materials and methods

### Zebrafish husbandry

**Fig. 1:**
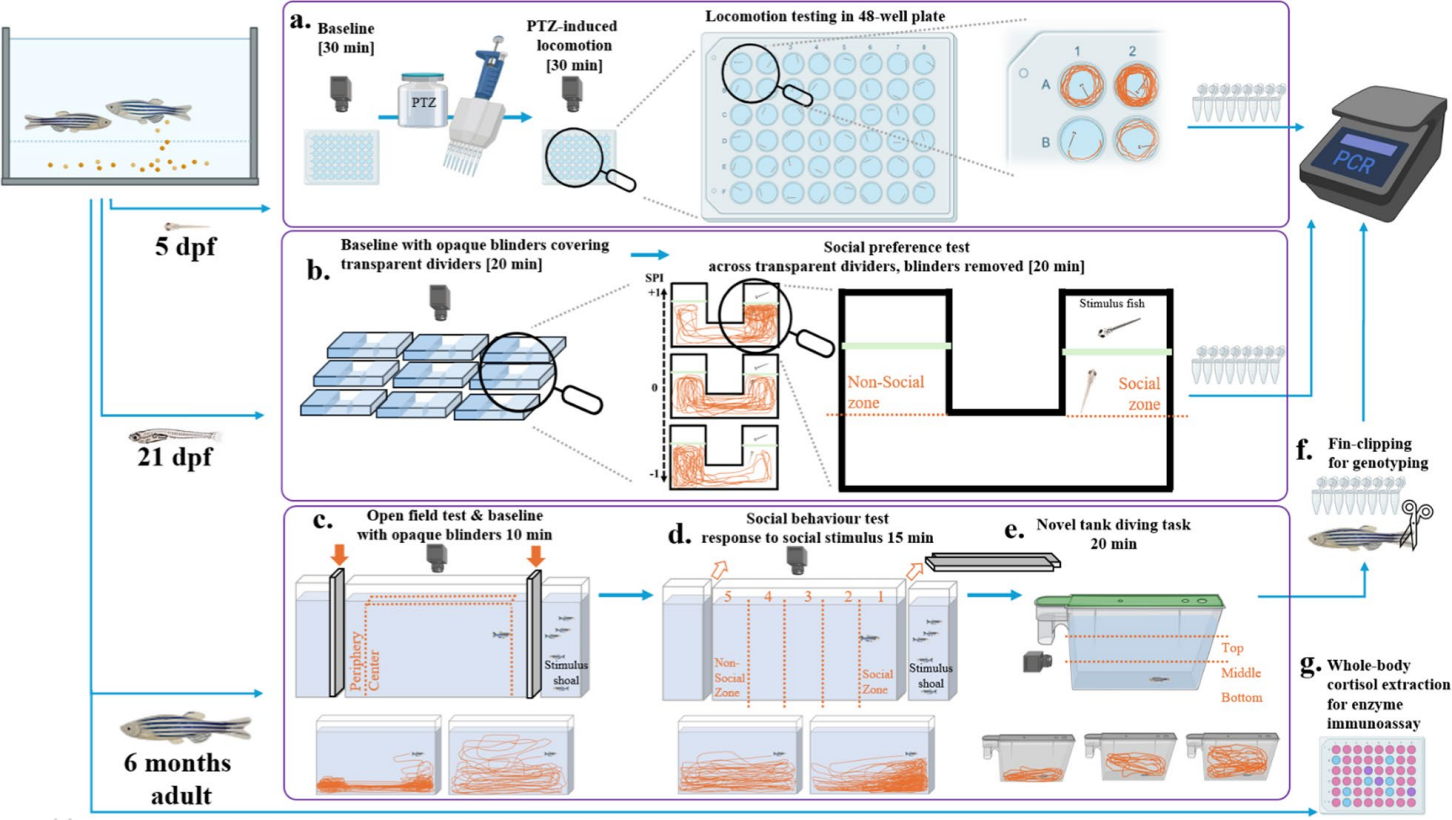
Schematic representation of the experimental protocol at 5 days post-fertilization (dpf), 21 dpf, and 6-months old adult fish. Experiments done at the three ages are separated and denoted with purple outline. Blue arrows show timeline of procedures while orange dotted lines represent virtual division of testing arenas into relevant areas. For 5 dpf larval fish (**a**), general locomotion was observed during a baseline period, followed by PTZ-induced hyperlocomotion. Representative examples of behavioural traces are provided to illustrate typical locomotion (wells A1 and B2), immobility (well B1), and hyperlocomotion (well A2). For 21 dpf fish (**b**), three traces illustrate preference (top), indifference (middle), and aversion (bottom) to social stimuli. The dotted orange lines mark the social and non-social zone used for calculation of social preference index [SPI]. For adult fish, the dotted orange lines indicate the line between border and center during (**c**) baseline open field testing, five zones during social preference testing (**d**) with zone 1 being closest to the shoal [social zone] and zone 5 being the furthest away from the shoal during social behavioural testing, and the division of water column into vertical top, middle and bottom areas (**e**) during novel tank diving test. Representative traces below show bottom-dwelling vs. exploration in higher water column in the open-field, no preference vs. social preference during the social behaviour test, and exploration of bottom only, middle, and top areas of the novel tank. After the behavioural testing, all fish were (**f**) fin-clipped under anaesthesia for genotyping. Separate group of experimentally naïve adult fish were used to measure whole-body cortisol levels (**g**) using enzyme immunoassay

Zebrafish (*Danio rerio*) were bred and housed at the University of Gothenburg. Breeding adults (parents of test fish), experimental fish, and additional stock AB fish used as social stimuli were housed in an automated recirculation system (Aquaneering, San Diego, USA), supplied by deionized water, and supplemented with Pro Reef salt (Tropic Marin, Wartenberg, Germany) and NaHCO_3_ to maintain conductivity at 700–800 μS and pH between 7.2—7.4. Adults and larvae were held in the same room under a 14:10 h light–dark cycle (light on between 8 AM and 10 PM) and at temperature of 27–28 °C. Starting from 6 days post-fertilization (dpf), fish were moved from the incubator to the system and fed three times per day with age-appropriate granulated fry food (ZM-100, ZM-200, ZM-300, ZM Systems, Winchester, UK) and brine shrimp nauplii (*Artemia salina*, ZM Fish foods, Winchester, UK) from 10 dpf. Adult fish were fed brine shrimp nauplii once a day and granular pellets (ZM Fish Food, Winchester, UK) twice a day. Zebrafish were held at a density of approximately 30 embryos per Petri dish during 0—5 dpf, 10 fish per litre during larval period [6—21 dpf], and 3–5 fish per litre as juveniles and adults. Larval experimental work took place at 5 dpf or at 21 dpf, and all larvae were experimentally naïve. Adult behavioural testing and cortisol sampling was done at 6-months of age. Sample sizes were based on existing zebrafish behavioural studies of appropriate ages and our previous work [57, 64, 91]. A total of 672 experimental 5 dpf fish were used, 140 experimental fish (and 40 AB fish as social stimuli) were used for social preference testing at 21 dpf. Also, 66 experimental fish (with 20 AB fish as social stimuli) were employed for adult behavioural tasks and 12 fish were used for cortisol analysis. Adult experimental and stimuli fish and 21 dpf stimuli fish were re-used for other purposes (brood stock, social stimuli, other pilot work, etc.). An overview of the experimental protocol and example behavioural tracks are illustrated in Fig. 1.

### Ethical considerations

Experiments adhered strictly to a study protocol approved by the local animal ethics committee in Gothenburg and followed the animal use and care guidelines of the Swedish National Board for Laboratory Animals (Ethical permit # 5.8.18–08496/2018) and European Directive 2010/63/EU. Experiments were planned as per PREPARE guidelines (Norecopa: Norway's National Consensus Platform for the advancement of "the 3 Rs") and data are reported here according to the ARRIVE guidelines from the National Centre for the Replacement, Refinement & Reduction of Animals in Research of UK.

### *mecp2*^*−/−*^ zebrafish line maintenance and genotyping

The previously described *mecp2*^−/−^ zebrafish line carrying *mecp2*^*Q63X*^ mutation in a nacre background was used [64] as well as *mecp2*^+/+^ siblings from the same parents. Prior to this experimental work, this line had been appropriately outcrossed to ensure removal of any potential unintended mutations typically associated with ENU-mutagenesis [64]. Larvae from both heterozygous and homozygous crosses were used in experiments. Heterozygous parents [*mecp2*^+/−^ paired with *mecp2*^+/−^] generated offspring with all three genotypes while homozygous pairs (*mecp2*^+/+^ males bred with *mecp2*^+/+^ females; *mecp2*^−/−^ males with *mecp2*^−/−^ females) generated only homozygous offspring genotypes. Homozygous *mecp2*-null parents [*mecp2*^−/−^ X *mecp2*^−/−^] were used for in-crosses specifically to check for any confounding influence of maternally deposited wildtype *mecp2* transcripts or Mecp2 protein from heterozygous parents. Age-matched wildtype AB zebrafish were raised to be used as stimuli animals for the social experiments. After the behavioural testing was performed as described below, all experimental 5 dpf and 21 dpf larvae were euthanized and adult fish were anesthetized and fin-clipped using age-appropriate MS-222 concentrations. Larval fish and fin samples were sent to LGC Service Lab, UK for genotyping by Kompetitive Allele-Specific PCR (KASP) for all experiments (except for the cortisol experiments, genotyping PCR for those were done in-house, as described before [64].

### Pentylenetetrazole (PTZ) preparation and exposure, and locomotion assay for 5 dpf fish

Larval zebrafish of 5 dpf were used to examine if the *mecp2*^*Q63X*^ fish showed a different locomotor response after treatment with PTZ, compared to controls. The larvae were placed in 48-well plates with 1.6 ml of system water in each well. Their behaviour was then recorded for 30 min to serve as a baseline, using a video camera (Sony Handycam HDR-CX405) mounted above the well plate. PTZ stock solution (200 mM) was prepared on the same day as experimental testing was performed by dissolving PTZ (Sigma-Aldrich) in Milli-Q water. The stock solution was diluted into the system water to get additional PTZ-stocks and 0.5 ml of appropriate solutions were mixed with system water of the wells to get desired final concentrations [0, 1, 2.5, 5, 7.5, and 10 mM]. The final concentrations used were well-established and based on prior zebrafish studies that showed hyperlocomotion and induction of seizures (Stage I and II) in larval zebrafish [92]. Following addition of PTZ, behaviour during PTZ exposure was recorded 30 min. Immediately after the experiments, the animals were euthanized in 300 mg/l MS-222 solution, placed in individual PCR tubes on dry ice, and stored at −80 degrees until sent for KASP genotyping (see above). Recorded videos of baseline and PTZ-treated behaviour were analyzed offline using automated tracking functions of EthoVision (Noldus Information Technology, Wageningen, Netherlands) software.

### Larval social behaviour assay for 21 dpf fish

Larval zebrafish of 21 dpf were used to investigate if young *mecp2*^*Q63X*^ fish displayed different social preferences compared to controls, at the time-point when social behaviour first becomes observable. The social preference of *mecp2*^+/+^, *mecp2*^+/−^ and *mecp2*^−/−^ zebrafish larvae was examined by placing them individually in a U-shaped test arena (3.5 × 4.5 cm) with a transparent glass bottom, as previously described [57, 91], placed on top of a light table and filmed from above using a video camera (Sony Handycam HDR-CX405). The test arenas had at each end a squared stimulus chamber which was separated from the test chamber by a wall of transparent glass. The stimulation chambers could be visually shielded from the test chamber by blinders made of opaque white plastic [57, 91].

The experiment started when the experimental fish were placed in the test chamber, visually shielded by blinders from the social stimulus, which consisted of one AB fish of the same age. In each experiment 16–18 test fish were run in parallel, in test chambers that were visually separated [i.e., test fish could not see other test fish, stimuli, or the experimenters]. Fish were recorded for 20 min during baseline open-exploration, and for 20 min after the opaque blinders were removed and social stimulus was visibly available. Recorded videos of behaviour during baseline and social-stimulus conditions were analyzed offline using EthoVision (Noldus) automated tracking. A social preference index (SPI) was calculated; the time spent in the non-social zone was subtracted from the time spent in the social zone and the result was divided by the total time [57, 91]. Fish (n = 3) that remained frozen for more than 50% of the time or did not enter both social and non-social zones during the baseline period were excluded from the analysis.

### Open field test for adult fish

The open field behaviour was observed in the baseline period of the first 10 min of the 6-months old adult social behaviour assay (described below) as adult *mecp2*^+*/*+^ and *mecp2*^*−/−*^ swim freely. The experimental set-up was comprised of one large test tank (60 cm × 30 cm), and two smaller side tanks (15 cm × 30 cm) that were positioned at the short ends of the test tank [91]. The water depth was 10 cm in both the test tank and in each of the side tanks. Tank water was made daily from deionized water and salts (Meersalt from Tropic Marin) and was pH and salinity adjusted to match the conditions of the home-tanks. The set-up was filmed from above using a video camera. Experimental tanks were visually isolated from surroundings and the experimenters using black non-transparent plastic sheets; additionally with opaque blinders ensuring that the test tank was visually isolated from the side tanks containing social stimuli fish during the baseline period [10 min]. To measure thigmotaxis behaviour from the recorded movies, a periphery zone was defined as a 5 cm distance from sides of the tank, leaving a 50 × 20 cm center zone in the middle of the chamber. Fish were tested individually, and the time spent in the defined periphery and center zones, along with total distances swam during the 10 min were quantified and compared between genotypes. Recorded videos were analyzed offline using TopScan behaviour analysis system (CleverSys Inc., Reston, Virginia, USA) software.

### Social behaviour assay for adult fish

Adult zebrafish were used to examine if *mecp2*^*−/−*^ fish exhibited atypical social response compared to control *mecp2*^+*/*+^ fish in adulthood. The stimulus shoal consisting of four age-matched AB fish was placed in one of the two side tanks whilst the second tank remained empty, and the shoal side was counterbalanced during trials. For the data analysis in TopScan behaviour analysis system, the bottom of the test tank was virtually divided into five equally sized horizontal zones. The zone closest to the shoal was zone one [social zone] with zone five being the furthest away from the shoal. The focal fish was first exposed to the social behaviour test (25 min duration, i.e., 10 min baseline period and 15 min with social stimuli), then it was gently netted to the novel tank diving set up.

### Novel tank diving test for adult fish

A subset of adult zebrafish was used to investigate if *mecp2*^−/−^ fish exhibited anxiety-like behaviour in response to a new environment compared to controls. The novel tank diving paradigm used one 1.5 l trapezoidal test tank, measuring 15 cm height × 27 cm top × 23.5 cm bottom × 6 cm width. Tank water pH and salinity was adjusted to match the conditions of the home-tanks. The experiment was recorded by a side-view camera for 20 min, using the program Debut Video Capture Software. Recorded videos were analysed offline using TopScan analysis system. During analysis, the test tank was virtually divided into 3 vertical zones, to denote top, middle, and bottom areas of the tank. At the end of the experimental day, a fin clip was taken from each fish during anesthesia with 164 mg/l MS-222 for genotyping. Post-clipping fish were kept individually in separate tanks for 24 h before returning to their home tank, and then used as brood stock once fins were healed.

### Cortisol sampling and extraction in adult fish

Whole-body cortisol levels were measured from mixed-sex and equally-sized zebrafish that were experimentally naïve and left undisturbed prior to sampling. Cortisol was extracted in three independent experiments from six adult *mecp2*^−/−^ and six control *mecp2*^+/+^ zebrafish as described previously [93].Cortisol was extracted in three independent experiments from six adult *mecp2*^−/−^ and six control *mecp2*^+/+^ zebrafish as described previously [93]. Briefly, each body sample was collected, weighed, and then dissected on ice into sections for homogenization. Each sample was mixed with 5.0 ml of diethyl ether, vortexed for 1 min, and centrifuged at 3500 rpm for 5 min. Once the ether was evaporated, the lipid cortisol residue was reconstituted in 1.0 ml of PBS. The extract was assayed for cortisol following manufacturer’s guidelines for high-sensitivity salivary cortisol enzyme immunoassay kit (Salimetrics, PA, USA < Catalogue no. 1–3002). Samples, standards, and controls were run in triplicates and reaction intensity was measured using a plate reader. Cortisol calculations (ng/g) were standardized to body weight and buffer volume for individual fish.

### Statistics and data analysis

Larval and juvenile behaviour videos were tracked using offline automated functions in EthoVision XT version 14 (Noldus) software while the adult behaviour videos were tracked using TopScan (CleverSys) offline automated tracking. Statistical analyses were performed using IBM SPSS statistics version 25 and group differences were considered significant when *p* < 0.05. Generally, after considering normality and homogeneity of variances, hypothesis testing on multiple group experimental design experiments was done using linear models (ANOVAs or Wilcoxon/Kruskal–Wallis tests) followed by suitable post-hoc contrasts if appropriate. Data were analyzed for main effects of genotype and PTZ-concentration [between-subject factors] and time [before and after PTZ-treatment or social stimulus as within-subject factor] as appropriate. Two-way ANOVAs were used for evaluation of effects of genotype and breeding [homozygous vs heterozygous parents], followed by Tukey’s multiple comparisons tests. Repeated measure ANOVAs were used for comparisons of mean values from multiple groups or time points. Unpaired or nested t-tests were used for comparisons of mean values from two genotype groups, as appropriate. If a main effect or interaction was found, post-hoc tests (t-tests, one-way ANOVAs, or Dunn's multiple comparisons test) were carried out for direction of effect, as applicable. As sex cannot be determined in larval fish at 5 dpf and 21 dpf, we examined only adult data for any potential sex differences, and when no sex-effects existed, male and female data were pooled together for analysis. To test null-hypotheses regarding differences between *mecp2*^+/+^ and *mecp2*^−/−^ fish, we acknowledge well-established and reproducible main effects of time/habituation and PTZ concentrations briefly and focused primarily on reporting only main effects of genotype and all significant interactions involving genotype.

## Results

### Five dpf ***mecp2***^−/−^ fish displayed hypolocomotion, but no difference in PTZ-induced hyperlocomotion

**Fig. 2:**
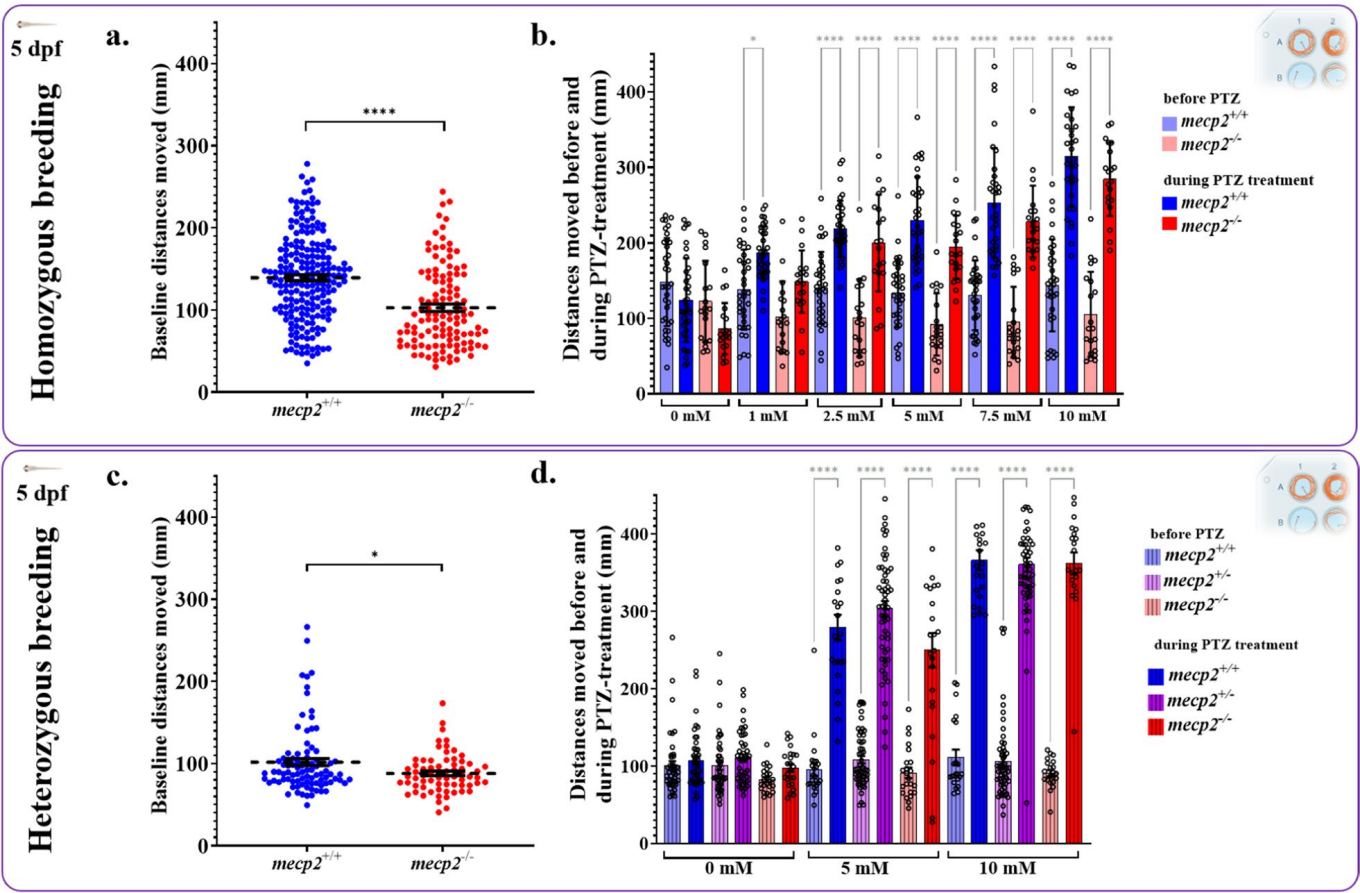
Larval locomotion behaviour was similarly affected in *mecp2*^*−/−*^ fish bred from homozygous parents (top panel) and heterozygous parents (bottom panel). **a** Baseline locomotion prior to PTZ-treatment for 5 dpf larvae from homozygous breeding showed that *mecp2*^*−/−*^ (n = 117) larvae moved significantly shorter distances than *mecp2*^+*/*+^ (n = 203). **b** Distances moved by *mecp2*^+*/*+^ (blue; n = 33–35) and *mecp2*^*−/−*^ (red; n = 19–20) fish from homozygous parents were similar during baseline (20 min before; lighter colours) and showed similar increases during PTZ-treatment (20 min, darker colours). **c** Baseline locomotion prior to PTZ-treatment for 5 dpf larvae from heterozygous *mecp2*^+/−^ crosses showed that *mecp2*^*−/−*^ (n = 68) larvae moved significantly shorter distances than *mecp2*^+*/*+^ (n = 90). **d** The distances moved were similar during baseline (20 min before; lighter colours) and the hyperlocomotion during PTZ-treatment (20 min, darker colours) was also similar in 5 dpf *mecp2*^+*/*+^, *mecp2*^+/−^, and *mecp2*^+*/*+^ fish derived from heterozygous breeding. Sample sizes were *mecp2*^+*/*+^ (blue; n = 41–65), *mecp2*^+/−^ (purple; n = 86–90) and *mecp2*^*−/−*^ (red; n = 35–37). Significant effects of PTZ-treatment within genotypes are indicated in grey. ****p < 0.0001 and *p < 0.05. Mean values shown as dotted lines and brackets mark SEM (a and c) and bars correspond to mean ± SEM (b and d)

Locomotion of 5 dpf larvae was tested in two sets of fish, offspring from homozygous parents (*mecp2*^*+/+*^ males bred with *mecp2*^+/+^ females; *mecp2*^−/−^ males with *mecp2*^−/−^ females; resulting in only two homozygous genotypes) and from heterozygous parents (*mecp2*^+/−^ X *mecp2*^+/−^; resulting in all three genotypes). Homozygous in-crosses control for any confounding effects of maternally deposited wildtype *mecp2* transcripts but has a drawback of comparing non-siblings. Conversely, siblings from heterozygous crosses provide control for any clutch/family differences but all embryos acquire maternally deposited *mecp2* [63]. When testing 5 dpf larvae from homozygous breeding, we found *mecp2*^*−/−*^ fish moved significantly less (Fig. 2a; t = 6.142, df = 318, p < 0.0001) than *mecp2*^+/+^ fish, and although five gradually higher concentrations of PTZ (0, 1, 2.5, 5, 7.5, 10 mM) increased locomotion in a dose-dependent way (p < 0.05, for main effect of PTZ), there were no differences between *mecp2*^+/+^ and *mecp2*^*−/−*^ fish in PTZ-induced hyperlocomotion (Fig. 2b; p > 0.05, for all PTZ concentrations). Likewise, the *mecp2*^−/−^ fish from heterozygous parents also moved shorter distances (Fig. 2c; t = 2.448, df = 156, p = 0.016) before PTZ-treatment compared to their *mecp2*^+*/*+^ siblings, and these fish were comparable to fish from homozygous crosses. While PTZ (control and two concentrations: 0, 5, and 10 mM) increased locomotion (Fig. 2d; p < 0.05, for main effect of PTZ) in all 5 dpf sibling larvae from heterozygous parents, there were no main effects of genotype on PTZ-induced hyperlocomotion (Fig. 2d; p < 0.05). Statistically significant differences in movement within genotypes between baseline and PTZ-treatment are presented in Table 2. Overall, these results show that *mecp2*^*−/−*^ zebrafish at 5 dpf, from both homozygous and heterozygous crosses, displayed hypolocomotion at baseline but reacted similarly to PTZ treatment as compared to the control groups.

**Table 2:** Within genotype comparisons for movement during baseline and PTZ treatment periods for various concentration

Homozygous breeding	PTZ	Genotype		Paired t-test	p-value
	0 mM	*mecp2*^+/+^		t = 1.760, df = 68	0.083
		*mecp2*^−/−^		t = 2.460, df = 36	0.0818
	1 mM	*mecp2*^+/+^		t = 4.486, df = 66	< 0.0001
		*mecp2*^−/−^		t = 3.257, df = 36	0.0025
	2.5 mM	*mecp2*^+/+^		t = 7.546, df = 66	< 0.0001
		*mecp2*^−/−^		t = 5.279, df = 36	< 0.0001
	5 mM	*mecp2*^+/+^		t = 7.704, df = 66	< 0.0001
		*mecp2*^−/−^		t = 7.783, df = 38	< 0.0001
	7.5 mM	*mecp2*^+/+^		t = 8.141, df = 64	< 0.0001
		*mecp2*^−/−^		t = 8.916, df = 38	< 0.0001
	10 mM	*mecp2*^+/+^		t = 10.96, df = 64	< 0.0001
		*mecp2*^−/−^		t = 10.77, df = 38	< 0.0001
Heterozygous breeding	0 mM	*mecp2*^+/+^		t = 0.8633, df = 86	0.3904
		*mecp2*^+/−^		t = 1.264, df = 90	0.2096
		*mecp2*^−/−^		t = 2.522, df = 46	0.0512
	5 mM	*mecp2*^+/+^		t = 10.05, df = 46	< 0.0001
		*mecp2*^+/−^		t = 17.83, df = 112	< 0.0001
		*mecp2*^−/−^		t = 6.884, df = 44	< 0.0001
	10 mM	*mecp2*^+/+^		t = 15.60, df = 42	< 0.0001
		*mecp2*^+/−^		t = 22.15, df = 112	< 0.0001
		*mecp2*^−/−^		t = 18.93, df = 40	< 0.0001

Zebrafish from both homozygous and heterozygous parental pairs responded similarly to increasing concentration of convulsive stimulant and displayed hyperlocomotion during PTZ-treatment

### *mecp2*^−/−^ zebrafish larvae do not display altered social preference at 21 dpf

**Fig. 3:**
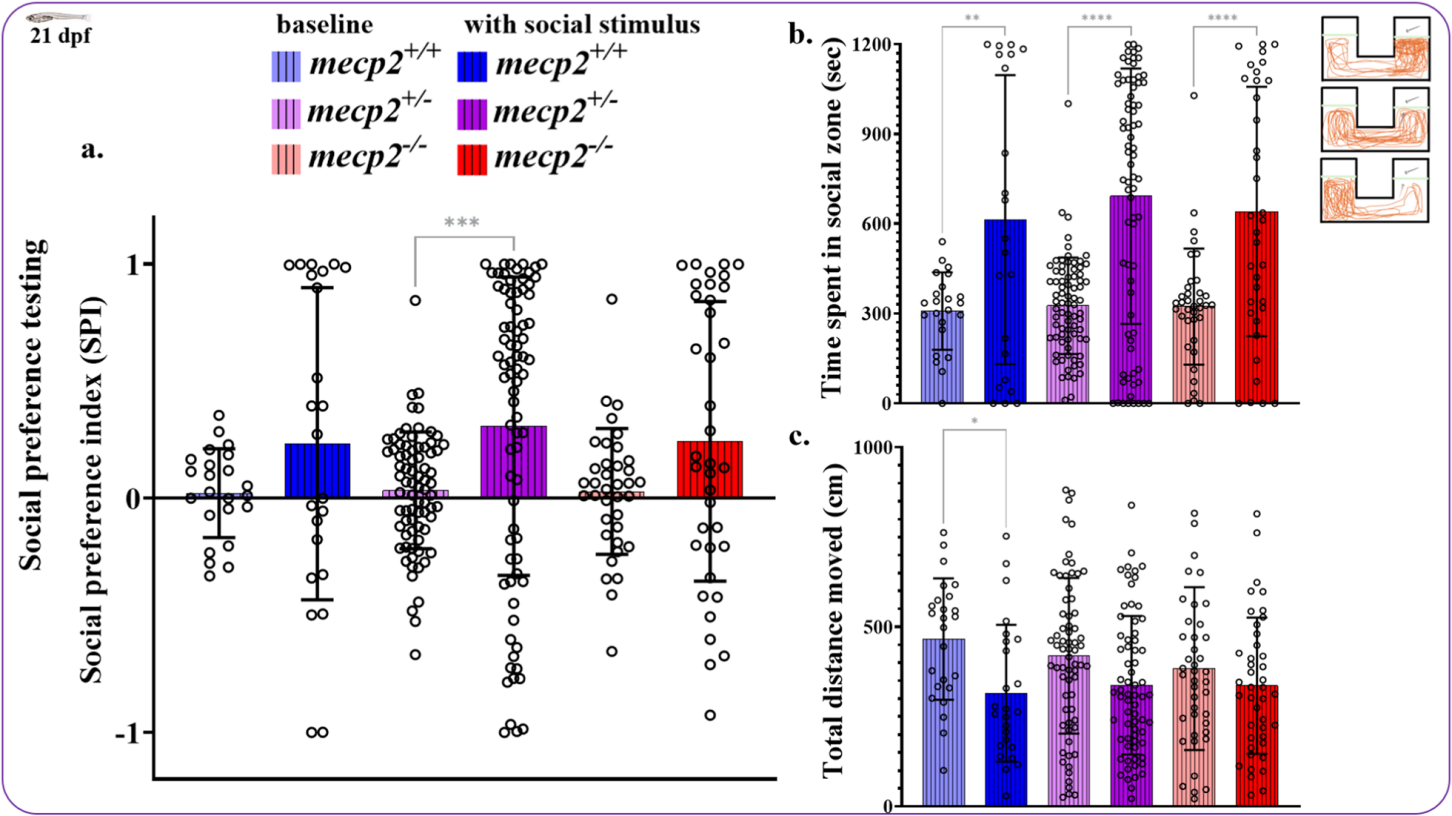
Social preference index, time spent in the social zone, and total distance moved by 21 dpf zebrafish during baseline and social stimulus phases. Social preference (**a**) was not altered in *mecp2* (n = 77) or *mecp2*^*−/−*^ (n = 37) larvae compared to *mecp2*^+*/*+^ (n = 23) zebrafish. Additionally, sibling zebrafish of all three genotypes spent similar length of time in the social zone (**b**) and swam similar distances (**c**) during the baseline and stimulus phases of the experiment. ****p < 0.0001 ***p < 0.001 **p < 0.01 and *p < 0.05. Mean ± SEM

To determine any effects of *mecp2* null-mutation on social preference and locomotion in three-week old fish, *mecp2*^−/−^ and *mecp2*^+/−^ zebrafish were compared to wildtype *mecp2*^+*/*+^ siblings from heterozygous breeding during baseline and social phases. The SPI distributions for social phase were not normal and non-parametric tests were used for determining the effect of genotype and visual social access. As illustrated in Fig. 3a, Kruskal–Wallis tests showed no main effect of genotype during baseline (p = 0.702) and social (0.922) phases. Wilcoxon signed rank test revealed that heterozygous *mecp2*^+/−^ fish (p < 0.001) showed significantly stronger bias for the zone adjacent to social stimulus (p = 0.140 for *mecp2*^+/+^; p = 0.0816 for *mecp2*^−/−^).

We also compared time spent in social zone separately as well and all three groups displayed stronger bias towards social zone in the social phase of the test. No main effect of genotype was detected (Fig. 3b; p > 0.05 for both baseline and social phase; Kruskal–Wallis test) and significantly more time was spent in the adjacent social zone during visual social access by for *mecp2*^+/+^, *mecp2*^+/−^, and *mecp2*^−/−^ (Fig. 3b; p = 0.009, p < 0.0001, and p < 0.0001, respectively; Wilcoxon signed ranks tests). Lastly, we analyzed total distances moved during baseline and social phases of the social preference test and a repeated-measures ANOVA showed a significant decrease in overall movement during visual access to social stimulus (Fig. 3c; F (1,132) = 24.735, p < 0.001) but no main effect or interaction of genotype on distances moved. Overall, zebrafish at 21 dpf displayed similar levels of locomotion and social preference regardless of their *mecp2* genotype.

### Anxiety behaviours in adult *mecp2*^−/−^ fish were affected but not open-field locomotion

**Fig. 4:**
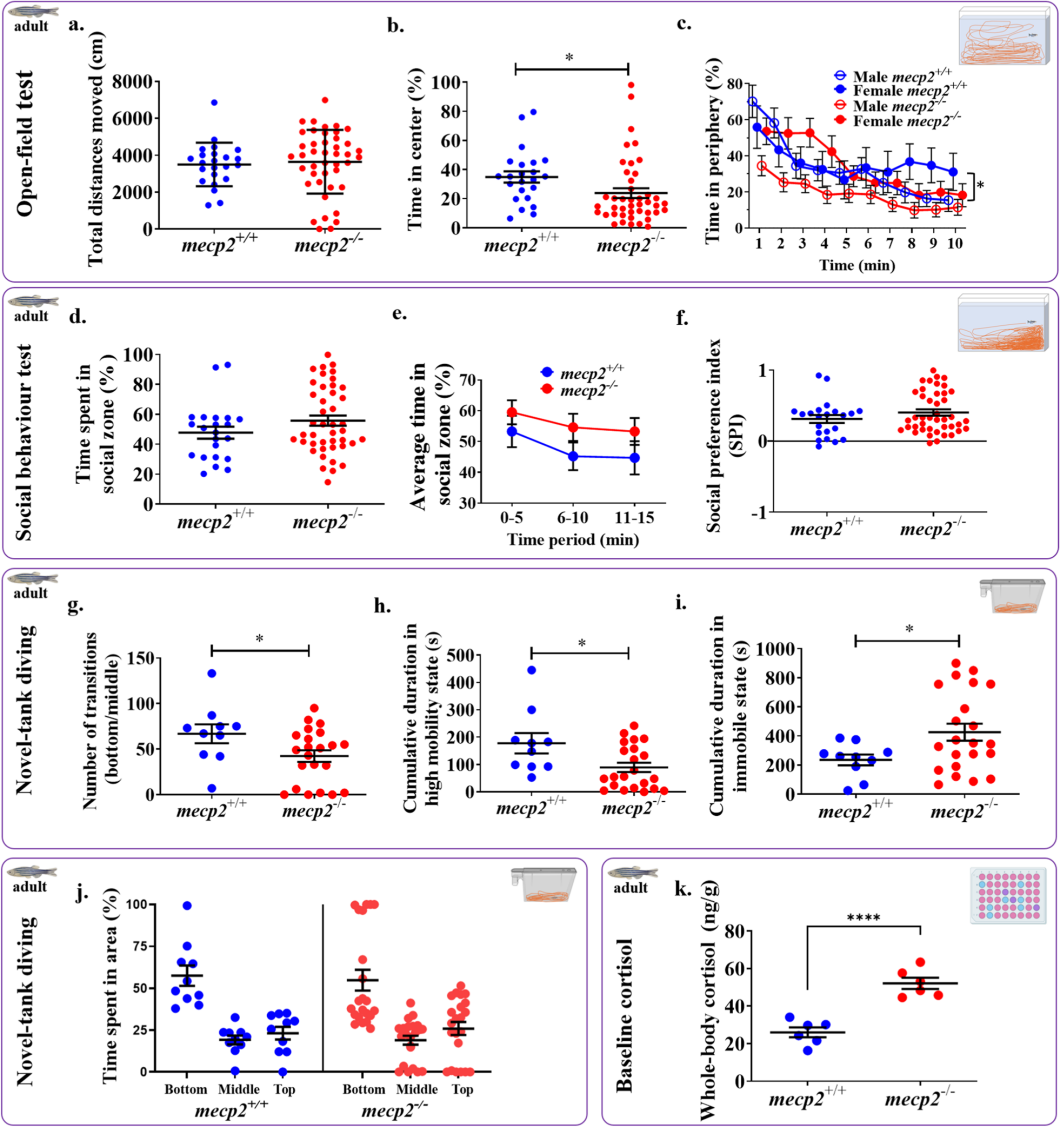
Anxiety-related behaviours and whole-body cortisol levels were altered in adult *mecp2*^*−/−*^ fish while locomotion and social preference were unaffected. Locomotion in the open-field (**a**) was not affected but adult *mecp2*^*−/−*^ fish spend significantly less time in the (**b**) centre zone in the open field compared to the *mecp2*^+*/*+^ group and (**c**) male *mecp2*^*−/−*^ fish showed somewhat less time in the periphery, compared to female *mecp2*^*−/−*^ fish. During the presentation of social stimuli, time spent in the social zone (**d**) was not significantly different in adult *mecp2*^*−/−*^ (n = 43) as compared to *mecp2*^*+/*+^ (n = 22). While *mecp2*^*−/−*^ fish generally trended towards more time closer to social stimuli than *mecp2*^*+/*+^ fish across the 15 min (**e**) the SPI was not different between the two groups of fish **(f**). In a novel tank diving task, the *mecp2*^*−/−*^ (n = 22) zebrafish made less bottom-to-middle transitions (**g**) and spent less time in high-mobility state (**h**) and more time in the immobile state **(i)** as compared to *mecp2*^+/+^ (n = 10) indicating an increase in bottom-dwelling and freezing behaviours, but overall time spent in bottom, middle, and top areas (**j**) was not different between the two genotypes. Baseline cortisol levels (**k**) were higher in experimentally naïve *mecp2*^*−/−*^ (n = 6) fish compared to *mecp2*^+*/*+^ (n = 6) zebrafish. *p < 0.05 and ****p < 0.0001. Mean ± SEM

To get a better understanding of movement and anxiety-related behaviours in adult *mecp2*^*−/−*^ fish, responses of 6-months old adult *mecp2*^*−/−*^ and *mecp2*^+*/*+^ fish individually placed in a large open-field were observed. Both genotype groups displayed similar locomotion and there was no main effect of genotype on total distances moved (Fig. 4a; p > 0.05; t-test). For analysis of thigmotaxis (wall-hugging) behaviour in adult *mecp2*^*−/−*^ fish, we also examined time spent in center vs. periphery of the testing tank. There was a main effect of genotype (Fig. 4b; t = 2.026, df = 64, p = 0.047) confirming that overall *mecp2*^*−/−*^ fish spent less time in the center zone during undisturbed observation. As indicated in Fig. 4c, all fish spent progressively more time in the center during the baseline, with male *mecp2*^*−/−*^ fish showed less thigmotaxis compared to female *mecp2*^*−/−*^ fish, and repeated-measures ANOVA showed a time x sex x genotype interaction (F (9, 558) = 3.418, p = 0.0004) and a significant effect of time (F (9, 558) = 20.98, p < 0.0001), but there were no main effects of genotype or sex for time spent in periphery. Thus, adult zebrafish displayed similar levels of locomotion regardless of their *mecp2* genotype whereas the *mecp2*^−/−^ fish displayed less time in the center zone indicating increased anxiety-like response compared to controls.

### *mecp2*^−/−^ zebrafish do not display altered social preference in adulthood

Both genotypes responded to social stimuli and spent similar length of time in the social zone when exposed to the conspecific shoals. We compared total time spent in social zone, time across the 15 min of social stimuli availability, and SPI, and found no differences between adult *mecp2*^*−/−*^ and *mecp2*^+*/*+^ (Fig. 4d, e, f; p > 0.05; t-tests). Overall, parallel to three-week-old zebrafish, adult fish also displayed similar levels of social preference regardless of their *mecp2* genotype.

### Adult *mecp2*^−/−^ zebrafish display increased anxiety behaviour in a novel tank

In the novel tank diving paradigm, we found a reduction in the number of bottom-to-middle transitions made by the *mecp2*^−/−^ fish (Fig. 4g; t = 2.080, df = 30, p = 0.0461). We also found that the duration of time in high mobility state was significantly reduced (Fig. 4h; t = 2.502, df = 30, p = 0.0181) and time being immobile was significantly increased (Fig. 4i; t = 2.093, df = 30, p = 0.0449) in the *mecp2*^−/−^ group compared to the *mecp2*^+/+^ fish, even while time spent in bottom, middle, and top sections of the novel tank did not differ between the genotypes (Fig. 4j; t = 0.0014, df = 4, p = 0.994; nested t-test). Taken together, these results indicate that adult *mecp2*^−/−^ zebrafish displayed elevated levels of anxiety behaviours, such as bottom-dwelling and freezing.

### Adult *mecp2*^−/−^ zebrafish have higher cortisol levels

Lacking functional *mecp2* affected resting whole-body cortisol levels in experimentally naïve unstressed fish. Compared to *mecp2*^+/+^ fish, similarly sized *mecp2*^−/−^ fish had higher cortisol levels (Fig. 4k; t = 6.512, df = 10, p < 0.0001) in their bodies. Thus, cortisol levels were elevated in adult *mecp2*^−/−^ zebrafish compared to controls.

## Discussion

In the current study of the zebrafish *mecp2* gene, we replicated the previous finding that *mecp2*^*Q63X*^ zebrafish larvae exhibit hypolocomotion compared to *mecp2*^+*/*+^ fish [64]. Our results also revealed that the GABA_A_ receptor antagonist PTZ induced hyperlocomotion similarly in *mecp2*^*−/−*^, *mecp2*^+/−^, and *mecp2*^+*/*+^ siblings. To our knowledge, this is the first analysis of social and anxiety behaviour and cortisol levels of adult *mecp2*^*Q63X*^ zebrafish. We found that *mecp2*^*−/−*^ zebrafish moved less and displayed higher anxiety behaviours and higher cortisol levels compared to *mecp2*^+*/*+^ fish. Interestingly, our results also revealed that *mecp2*^*−/−*^ fish showed similar social preference as *mecp2*^+*/*+^, both during ontogeny of social behaviour at 21 dpf larvae and as mature form of adulthood socialization.

Recently, several zebrafish mutant lines lacking genes linked to neurodevelopmental disorders have shown phenotypic seizure-like behaviour [92, 94–97], social dysfunction [86, 98, 99] and locomotion abnormalities as well as changes in activity and morphology of relevant brain regions [96, 100]. Previously, zebrafish *mecp2* gene manipulation studies have showed recapitulation of milder Rett syndrome phenotypes in mutants and morphants, such as motor abnormalities and changes in larval anxiety behaviour [64] and transcriptomic and proteomic analyses have suggested impaired visual function and energy metabolism in *mecp2*^*Q63X*^ fish [66, 67]. Exposure to genotoxic and environmental regulators such as morphine [77], alcohol [69], chronic BPA [74], crude oil and lead [75] have also been shown to regulate *mecp2* expression and Mecp2 function in zebrafish. Altogether, these studies have further increased value of zebrafish models of Rett syndrome and provided additional impetus to characterize motor, social, stress, and anxiety-like behaviour in developmental and adult *mecp2*^*Q63X*^ fish.

### Locomotion profile of *mecp2*^*Q63X*^ fish

Similar to the current study, previously loss-of-function of *mecp2* has been reported to cause hypolocomotion in 5–6 dpf larval zebrafish [64]. Furthermore, morpholino-mediated knockdown of *mecp2* led to a decrease in motor activity as early as 28 hpf in zebrafish morphants [78]. The hypolocomotion phenotype in larval zebrafish was suggested to be caused by an increase in abnormal axonal branches of caudal primary motor neurons [78] whereas selective activation of cholinergic neurotransmission reversed the locomotor impairment in mice [101]. While we did not find any differences in locomotion of adult *mecp2*^*−/−*^ and *mecp2*^+*/*+^ fish in the open-field, higher levels of freezing and bottom-dwelling were seen in the novel tank test of adult *mecp2*^*−/−*^ fish. Here it is relevant to note that using the same null-mutation model and proteomics, impairments in energy metabolism, redox balance, and structure and function of cardiac and skeletal muscle in larval and adult fish have been suggested [66]. While our tests induced some anxiety for short durations, evaluation of *mecp2*^*−/−*^ fish in longer and more exhaustive motor tasks requiring more energy expenditure and higher muscle performance would shed light on behavioural consequences of altered proteomes.

### Stress and anxiety-related behaviours in *mecp2*^*Q63X*^ fish

The elevated anxiety behaviour and higher cortisol levels observed in the *mecp2*^*Q63X*^ zebrafish are of interest as these mimic frequently displayed heightened anxiety and mood fluctuation symptoms [10, 11, 19, 23, 24] and related aberrant HPA-axis function in Rett syndrome patients, including increased cortisol levels [19, 21–23]. Also, mouse Mecp2 has been reported to regulate stress vulnerability [46] and key genes in the HPA axis [47, 102, 103], and mice with truncated *Mecp2* allele have increased expression of corticotropin-releasing hormone and elevated levels of corticosterone in response to stress [104]. We purposely tested baseline cortisol levels in experimentally naïve fish as novel environments and handling during behavioural procedures can induce stress and led to changes in whole-body cortisol. While circulating levels of cortisol and corticosterone oscillate with circadian clock of the body and do not have a one-to-one relationship with psychological stress [105], higher resting cortisol levels observed in the *mecp2*^*Q63X*^ mutant fish provide evidence of altered stress function linked to *mecp2* null-mutation. Future *mecp2*^*Q63X*^ zebrafish studies with comparison of cortisol levels in resting and induced acute and chronic stress conditions, along with pharmacological treatments with anxiolytics and antidepressants would be valuable in further clarification of links between MECP2 deficiency and aberrant HPA-axis function in Rett syndrome patients.

In line with our results, *Mecp2* knockout mice have been reported to display increased anxiety-like behaviours [101, 104, 106], whereas Stearns et al. [107] found decreased anxiety-like behaviour in mutant *Mecp2* mice and Samaco et al. [47] showed that overexpression of Mecp2 also increases anxiety-related behaviours in mice. Thus, investigations of anxiety-related behaviours in animal models of Rett syndrome have so far yielded some inconsistent findings across as well as within species, but the association with increased stress vulnerability and atypical stress responsiveness are fairly established. Also, whereas we here show elevated anxiety when comparing adult *mecp2*^-/-^ zebrafish with *mecp2*^+/+^  fish of the same age, a previous study doing the same comparison of 6 dpf zebrafish, carrying the same null-mutation, instead showed decreased anxiety [assessed using thigmotaxis or wall-hugging] [64]. These results, along with the locomotion findings, demonstrate distinct effects of *mecp2* deletion in young and adult zebrafish, indicating that Mecp2 may regulate behaviours differently during early development and adulthood.

### Vision-based social behaviour in *mecp2*^*Q63X*^ fish

In contrast to patients with Rett syndrome and mammalian models lacking functional Mecp2, our investigations did not reveal any substantial role of *mecp2* in the expression of social preference behaviour of 21 dpf *mecp2*^*−/−*^ larvae and adult *mecp2*^*−/−*^ fish. Adult *Mecp2* knockout mice display a hypersocial phenotype in several studies whereas juvenile play behaviour was reduced in female rats [32]. Aside from heightened anxiety mentioned above, the lack of differences in our social phenotype could also be a consequence of using behavioural tests that did not provide enough motivation or complexity of social interaction (for example, we used only one fish as social stimulus vs empty space for pfd 15 and visual-only access to a group of four conspecifics for adult fish). More stimulating social testing conditions may potentially disclose functional regulation of social behaviour by Mecp2 in zebrafish, such as larger shoals, isolation-induced social behaviour, interactive shoaling with live conspecifics fish where test fish would swim in a group, or social buffering during stress, etc., and higher complexity of social interaction (such as social memory or recognition, and dyadic interactions seen during aggression or mating). Nonetheless, the responses to vision-based social stimuli and novel environments in our study further suggest either no effects of lacking *mecp2* on vision or compensation by additional factors in older 21 dpf and adult fish observed. Future studies examining vision directly using higher-resolution observations and comprehensive investigations of shoaling, mating, and dyadic aggression dynamics in zebrafish *mecp2*^*Q63X*^ fish will help elucidate these interactions.

## Limitations

A large number of patients with Rett syndrome have intellectual disability and seizures. An obvious limitation of the current study is lack of cognitive evaluation of *mecp2*^*Q63X*^ fish and future zebrafish studies using this model and other Mecp2 manipulations should assess cognitive abilities in combination with social, motor and anxiety-like behaviours. Our results indicated that pharmacological inhibition of GABA receptors induced a hyperlocomotion response similarly in all fish independently of the presence of the Mecp2 protein. In contrast, *Mecp2* knockout mice display cortical discharges consistent with absence epilepsy [108] and loss of MECP2 specifically from forebrain excitatory neurons leads to cortical hyperexcitation and seizures [109], leading to suggestion of alterations of the balance between the excitatory and inhibitory neurotransmission. Thus, despite our finding, to fully exclude that *mecp2* exert action on GABA signalling, and to explore any effects on glutamatergic functioning, future zebrafish studies should include alternative convulsants and electrophysiological recordings across development.

Considering the apparent and devastating deficits seen in humans carrying deficient MECP2, the lack of phenotypical differences in sociability and PTZ-induced hyperlocomotion between *mecp2*^*−/−*^ and *mecp2*^+*/*+^ zebrafish are noticeable. Our methodology does not allow distinction between seizure stages and thus well-established hypolocomotion in *mecp2*^*−/−*^ fish could mask PTZ-induced changes. More comprehensive studies with electrophysiology and higher resolution observations are needed to confirm if the hypolocomotion is concealing the seizure-like activity or if the typical locomotion in PTZ-treated *mecp2*^*−/−*^ larvae could be due to other factors (motivation, energy, etc.). Taking in consideration the variability of symptoms seen in patients with Rett syndrome, we cannot rule out the possibility that behavioural variations may exist at specific or later stages of zebrafish development—as seen in other animal models of the syndrome [110]. Lastly, a caveat of using zebrafish models is that unlike humans, the zebrafish *mecp2* gene is not on X-chromosome and Sex determination in zebrafish is not influenced by sex chromosomes, but rather autosomal chromosomes and environmental factors play important roles. Thus, while viable and fertile *mecp2*^−/−^ fish of both sexes can be studied, translation value of conclusions from zebrafish studies alone may be limited.

## Conclusion

Dysfunction of MECP2, a protein vital for neuronal maturation and silencing of genes during growth and experience-dependent changes, is linked to Rett syndrome, autism and other neurodevelopmental disorders. Using a zebrafish model, we studied behaviour of young and adult zebrafish that lacked this protein and discovered that these fish mimic milder human symptoms of atypical movement and elevated anxiety, but overall vision and social behaviour were unaffected.

In conclusion, the current study showed that zebrafish Mecp2 modulates locomotion and anxiety but not sociability or PTZ-induced hyperlocomotion. While this is the first study to explore adult behaviour in *mecp2*^*−/−*^ zebrafish, we have essentially raised more questions than answered. Consequently, there is an urgent necessity to complement the existing ENU-derived and TILLING-selected line used in our experiments and create targeted mutant zebrafish models with greater precision. Conditional mutants that reduce or eradicate Mecp2 function at specific developmental timepoints or in specific brain areas and neuron-types during notable experiences (such as socialization or when exposed to stressful stimuli) can further help elucidate how experience-dependent neuronal changes are regulated by zebrafish Mecp2. Zebrafish research has grown exponentially with advancements in gene editing techniques, such as CRISPR-mediated base- and prime-editing, RNA-editing, and combining brain-specific manipulation of *mecp2* with high-throughput studies can make understanding of Mecp2 function and potential therapeutic developments more feasible.

## Data Availability

The datasets generated during and/or analysed during the current study and any further information regarding materials are available from the corresponding author upon request.

## References

[1] Li CH, Coffey EL, Dall’Agnese A, Hannett NM, Tang X, Henninger JE, Platt JM, Oksuz O, Zamudio AV, Afeyan LK, et al. MeCP2 links heterochromatin condensates and neurodevelopmental disease. Nature. 2020;586(7829):440–4. 32698189 10.1038/s41586-020-2574-4 PMC7735819

[2] Bin Akhtar G, Buist M, Rastegar M. MeCP2 and transcriptional control of eukaryotic gene expression. Eur J Cell Biol. 2022;101(3): 151237. 35588541 10.1016/j.ejcb.2022.151237

[3] Amir RE, Van den Veyver IB, Wan M, Tran CQ, Francke U, Zoghbi HY. Rett syndrome is caused by mutations in X-linked MECP2, encoding methyl-CpG-binding protein 2. Nat Genet. 1999;23(2):185–8. 10508514 10.1038/13810

[4] Weaving LS, Ellaway CJ, Gécz J, Christodoulou J. Rett syndrome: clinical review and genetic update. J Med Genet. 2005;42(1):1–7. 15635068 10.1136/jmg.2004.027730 PMC1735910

[5] Bienvenu T, Carrié A, de Roux N, Vinet MC, Jonveaux P, Couvert P, Villard L, Arzimanoglou A, Beldjord C, Fontes M, et al. MECP2 mutations account for most cases of typical forms of Rett syndrome. Hum Mol Genet. 2000;9(9):1377–84. 10814719 10.1093/hmg/9.9.1377

[6] Nan X, Ng HH, Johnson CA, Laherty CD, Turner BM, Eisenman RN, Bird A. Transcriptional repression by the methyl-CpG-binding protein MeCP2 involves a histone deacetylase complex. Nature. 1998;393(6683):386–9. 9620804 10.1038/30764

[7] Wakefield RI, Smith BO, Nan X, Free A, Soteriou A, Uhrin D, Bird AP, Barlow PN. The solution structure of the domain from MeCP2 that binds to methylated DNA. J Mol Biol. 1999;291(5):1055–65. 10518942 10.1006/jmbi.1999.3023

[8] Klose RJ, Bird AP. MeCP2 behaves as an elongated monomer that does not stably associate with the Sin3a chromatin remodeling complex. J Biol Chem. 2004;279(45):46490–6. 15322089 10.1074/jbc.M408284200

[9] Rett A. On a unusual brain atrophy syndrome in hyperammonemia in childhood. Wien Med Wochenschr. 1966;116(37):723–6. 5300597

[10] Percy AK, Neul JL, Benke TA, Marsh ED, Glaze DG. A review of the Rett Syndrome Behaviour Questionnaire and its utilization in the assessment of symptoms associated with Rett syndrome. Front Pediatr. 2023;11:1229553. 37635789 10.3389/fped.2023.1229553 PMC10450502

[11] Raspa M, Gwaltney A, Bann C, von Hehn J, Benke TA, Marsh ED, Peters SU, Ananth A, Percy AK, Neul JL. Psychometric assessment of the Rett syndrome Caregiver assessment of symptom severity (RCASS). J Autism Dev Dis. 2024;23:185. 10.1007/s10803-024-06238-0 PMC11374935 38438817

[12] May DM, Neul J, Piña-Garza JE, Kponee-Shovein K, Satija A, Mahendran M, Downes N, Sheng K, Lema N, Boca A, et al. Gastrointestinal manifestations in pediatric and adult patients with Rett syndrome: an analysis of US claims and physician survey data. J Comp Eff Res. 2024;13(1): e230054. 37971297 10.57264/cer-2023-0054 PMC10842289

[13] Neul JL, Percy AK, Benke TA, Berry-Kravis EM, Glaze DG, Marsh ED, Lin T, Stankovic S, Bishop KM, Youakim JM. Trofinetide for the treatment of Rett syndrome: a randomized phase 3 study. Nat Med. 2023;29(6):1468–75. 37291210 10.1038/s41591-023-02398-1 PMC10287558

[14] Neul JL, Percy AK, Benke TA, Berry-Kravis EM, Glaze DG, Peters SU, Marsh ED, An D, Bishop KM, Youakim JM. Trofinetide treatment demonstrates a benefit over placebo for the ability to communicate in Rett syndrome. Pediatr Neurol. 2024;152:63–72. 38232652 10.1016/j.pediatrneurol.2023.11.005

[15] Khwaja OS, Ho E, Barnes KV, O’Leary HM, Pereira LM, Finkelstein Y, Nelson CA 3rd, Vogel-Farley V, DeGregorio G, Holm IA, et al. Safety, pharmacokinetics, and preliminary assessment of efficacy of mecasermin (recombinant human IGF-1) for the treatment of Rett syndrome. Proc Natl Acad Sci USA. 2014;111(12):4596–601. 24623853 10.1073/pnas.1311141111 PMC3970488

[16] Hagberg B, Aicardi J, Dias K, Ramos O. A progressive syndrome of autism, dementia, ataxia, and loss of purposeful hand use in girls: Rett’s syndrome: report of 35 cases. Ann Neurol. 1983;14(4):471–9. 6638958 10.1002/ana.410140412

[17] Hagberg B, Hanefeld F, Percy A, Skjeldal O. An update on clinically applicable diagnostic criteria in Rett syndrome. Comments to Rett syndrome clinical criteria consensus panel satellite to european paediatric neurology society meeting, Baden Baden, Germany, 11 september 2001. Eur J Paediatr Neurol. 2002;6(5):293–7. 12378695 10.1053/ejpn.2002.0612

[18] Neul JL, Kaufmann WE, Glaze DG, Christodoulou J, Clarke AJ, Bahi-Buisson N, Leonard H, Bailey ME, Schanen NC, Zappella M, et al. Rett syndrome: revised diagnostic criteria and nomenclature. Ann Neurol. 2010;68(6):944–50. 21154482 10.1002/ana.22124 PMC3058521

[19] Buchanan CB, Stallworth JL, Joy AE, Dixon RE, Scott AE, Beisang AA, Benke TA, Glaze DG, Haas RH, Heydemann PT, et al. Anxiety-like behavior and anxiolytic treatment in the Rett syndrome natural history study. J Neurodev Disord. 2022;14(1):31. 35568815 10.1186/s11689-022-09432-2 PMC9107202

[20] Tarquinio DC, Hou W, Neul JL, Kaufmann WE, Glaze DG, Motil KJ, Skinner SA, Lee HS, Percy AK. The changing face of survival in Rett syndrome and MECP2-related disorders. Pediatr Neurol. 2015;53(5):402–11. 26278631 10.1016/j.pediatrneurol.2015.06.003 PMC4609589

[21] Motil KJ, Schultz RJ, Abrams S, Ellis KJ, Glaze DG. Fractional calcium absorption is increased in girls with Rett syndrome. J Pediatr Gastroenterol Nutr. 2006;42(4):419–26. 16641581 10.1097/01.mpg.0000189370.22288.0c

[22] Echenne B, Bressot N, Bassir M, Daures JP, Rabinowitz A. Cerebrospinal fluid beta-endorphin and cortisol study in Rett syndrome. J Child Neurol. 1991;6(3):257–62. 1875029 10.1177/088307389100600310

[23] Byiers BJ, Payen A, Feyma T, Panoskaltsis-Mortari A, Ehrhardt MJ, Symons FJ. Associations among diurnal salivary cortisol patterns, medication use, and behavioral phenotype features in a community sample of Rett syndrome. Am J Intellect Dev Disabil. 2020;125(5):353–68. 32936892 10.1352/1944-7558-125.5.353 PMC10699094

[24] Buchanan CB, Stallworth JL, Scott AE, Glaze DG, Lane JB, Skinner SA, Tierney AE, Percy AK, Neul JL, Kaufmann WE. Behavioral profiles in Rett syndrome: data from the natural history study. Brain Dev. 2019;41(2):123–34. 30217666 10.1016/j.braindev.2018.08.008 PMC6392009

[25] Achilly NP, He LJ, Kim OA, Ohmae S, Wojaczynski GJ, Lin T, Sillitoe RV, Medina JF, Zoghbi HY. Deleting Mecp2 from the cerebellum rather than its neuronal subtypes causes a delay in motor learning in mice. eLife. 2021;10:e64833. 10.7554/eLife.64833 PMC7837679 33494858

[26] Chen RZ, Akbarian S, Tudor M, Jaenisch R. Deficiency of methyl-CpG binding protein-2 in CNS neurons results in a Rett-like phenotype in mice. Nat Genet. 2001;27(3):327–31. 11242118 10.1038/85906

[27] Guy J, Hendrich B, Holmes M, Martin JE, Bird A. A mouse Mecp2-null mutation causes neurological symptoms that mimic Rett syndrome. Nat Genet. 2001;27(3):322–6. 11242117 10.1038/85899

[28] Kishi N, MacDonald JL, Ye J, Molyneaux BJ, Azim E, Macklis JD. Reduction of aberrant NF-κB signalling ameliorates Rett syndrome phenotypes in Mecp2-null mice. Nat Commun. 2016;7:10520. 26821816 10.1038/ncomms10520 PMC4740176

[29] Pelka GJ, Watson CM, Radziewic T, Hayward M, Lahooti H, Christodoulou J, Tam PP. Mecp2 deficiency is associated with learning and cognitive deficits and altered gene activity in the hippocampal region of mice. Brain. 2006;129(Pt 4):887–98. 16467389 10.1093/brain/awl022

[30] Ribeiro MC, MacDonald JL. Sex differences in Mecp2-mutant Rett syndrome model mice and the impact of cellular mosaicism in phenotype development. Brain Res. 2020;1729: 146644. 31904347 10.1016/j.brainres.2019.146644 PMC7024565

[31] Bhattacherjee A, Mu Y, Winter MK, Knapp JR, Eggimann LS, Gunewardena SS, Kobayashi K, Kato S, Krizsan-Agbas D, Smith PG. Neuronal cytoskeletal gene dysregulation and mechanical hypersensitivity in a rat model of Rett syndrome. Proc Natl Acad Sci USA. 2017;114(33):E6952-e6961. 28760966 10.1073/pnas.1618210114 PMC5565404

[32] Veeraragavan S, Wan YW, Connolly DR, Hamilton SM, Ward CS, Soriano S, Pitcher MR, McGraw CM, Huang SG, Green JR, et al. Loss of MeCP2 in the rat models regression, impaired sociability and transcriptional deficits of Rett syndrome. Hum Mol Genet. 2016;25(15):3284–302. 27365498 10.1093/hmg/ddw178 PMC5179927

[33] Liu Z, Zhou X, Zhu Y, Chen ZF, Yu B, Wang Y, Zhang CC, Nie YH, Sang X, Cai YJ, et al. Generation of a monkey with MECP2 mutations by TALEN-based gene targeting. Neurosci Bull. 2014;30(3):381–6. 24838303 10.1007/s12264-014-1434-8 PMC5562616

[34] Kim KY, Hysolli E, Park IH. Neuronal maturation defect in induced pluripotent stem cells from patients with Rett syndrome. Proc Natl Acad Sci USA. 2011;108(34):14169–74. 21807996 10.1073/pnas.1018979108 PMC3161557

[35] Li Y, Wang H, Muffat J, Cheng AW, Orlando DA, Lovén J, Kwok SM, Feldman DA, Bateup HS, Gao Q, et al. Global transcriptional and translational repression in human-embryonic-stem-cell-derived Rett syndrome neurons. Cell Stem Cell. 2013;13(4):446–58. 24094325 10.1016/j.stem.2013.09.001 PMC4053296

[36] Marchetto MC, Carromeu C, Acab A, Yu D, Yeo GW, Mu Y, Chen G, Gage FH, Muotri AR. A model for neural development and treatment of Rett syndrome using human induced pluripotent stem cells. Cell. 2010;143(4):527–39. 21074045 10.1016/j.cell.2010.10.016 PMC3003590

[37] Mori M, Yoshii S, Noguchi M, Takagi D, Shimizu T, Ito H, Matsuo-Takasaki M, Nakamura Y, Takahashi S, Hamada H, et al. Generation of human induced pluripotent stem cell lines derived from four Rett syndrome patients with MECP2 mutations. Stem Cell Res. 2024;77: 103432. 38703668 10.1016/j.scr.2024.103432

[38] Rodrigues DC, Mufteev M, Yuki KE, Narula A, Wei W, Piekna A, Liu J, Pasceri P, Rissland OS, Wilson MD, et al. Buffering of transcription rate by mRNA half-life is a conserved feature of Rett syndrome models. Nat Commun. 2023;14(1):1896. 37019888 10.1038/s41467-023-37339-6 PMC10076348

[39] Bahram Sangani N, Koetsier J, Gomes AR, Diogo MM, Fernandes TG, Bouwman FG, Mariman ECM, Ghazvini M, Gribnau J, Curfs LMG, et al. Involvement of extracellular vesicle microRNA clusters in developing healthy and Rett syndrome brain organoids. Cell Mol Life Sci. 2024;81(1):410. 39305343 10.1007/s00018-024-05409-7 PMC11416455

[40] Mok RSF, Zhang W, Sheikh TI, Pradeepan K, Fernandes IR, DeJong LC, Benigno G, Hildebrandt MR, Mufteev M, Rodrigues DC, et al. Wide spectrum of neuronal and network phenotypes in human stem cell-derived excitatory neurons with Rett syndrome-associated MECP2 mutations. Transl Psychiatry. 2022;12(1):450. 36253345 10.1038/s41398-022-02216-1 PMC9576700

[41] Xu YJ, Liu PP, Yan ZZ, Mi TW, Wang YY, Li Q, Teng ZQ, Liu CM. KW-2449 and VPA exert therapeutic effects on human neurons and cerebral organoids derived from MECP2-null hESCs. Stem Cell Res Ther. 2022;13(1):534. 36575558 10.1186/s13287-022-03216-0 PMC9795779

[42] Trujillo CA, Adams JW, Negraes PD, Carromeu C, Tejwani L, Acab A, Tsuda B, Thomas CA, Sodhi N, Fichter KM, et al. Pharmacological reversal of synaptic and network pathology in human MECP2-KO neurons and cortical organoids. EMBO Mol Med. 2021;13(1): e12523. 33501759 10.15252/emmm.202012523 PMC7799367

[43] Dani VS, Chang Q, Maffei A, Turrigiano GG, Jaenisch R, Nelson SB. Reduced cortical activity due to a shift in the balance between excitation and inhibition in a mouse model of Rett syndrome. Proc Natl Acad Sci USA. 2005;102(35):12560–5. 16116096 10.1073/pnas.0506071102 PMC1194957

[44] Asaka Y, Jugloff DG, Zhang L, Eubanks JH, Fitzsimonds RM. Hippocampal synaptic plasticity is impaired in the Mecp2-null mouse model of Rett syndrome. Neurobiol Dis. 2006;21(1):217–27. 16087343 10.1016/j.nbd.2005.07.005

[45] Calfa G, Percy AK, Pozzo-Miller L. Experimental models of Rett syndrome based on Mecp2 dysfunction. Exp Biol Med. 2011;236(1):3–19. 10.1258/ebm.2010.010261 PMC3059199 21239731

[46] Cosentino L, Bellia F, Pavoncello N, Vigli D, D’Addario C, De Filippis B. Methyl-CpG binding protein 2 dysfunction provides stress vulnerability with sex- and zygosity-dependent outcomes. Eur J Neurosci. 2022;55(9–10):2766–76. 33655553 10.1111/ejn.15165

[47] Samaco RC, Mandel-Brehm C, McGraw CM, Shaw CA, McGill BE, Zoghbi HY. Crh and Oprm1 mediate anxiety-related behavior and social approach in a mouse model of MECP2 duplication syndrome. Nat Genet. 2012;44(2):206–11. 22231481 10.1038/ng.1066 PMC3267865

[48] Chao HT, Zoghbi HY, Rosenmund C. MeCP2 controls excitatory synaptic strength by regulating glutamatergic synapse number. Neuron. 2007;56(1):58–65. 17920015 10.1016/j.neuron.2007.08.018 PMC2198899

[49] Nelson ED, Bal M, Kavalali ET, Monteggia LM. Selective impact of MeCP2 and associated histone deacetylases on the dynamics of evoked excitatory neurotransmission. J Neurophysiol. 2011;106(1):193–201. 21511710 10.1152/jn.00751.2010 PMC3129735

[50] Nelson ED, Kavalali ET, Monteggia LM. MeCP2-dependent transcriptional repression regulates excitatory neurotransmission. Current Biol. 2006;16(7):710–6. 10.1016/j.cub.2006.02.062 16581518

[51] Gadalla KK, Bailey ME, Cobb SR. MeCP2 and Rett syndrome: reversibility and potential avenues for therapy. Biochem J. 2011;439(1):1–14. 21916843 10.1042/BJ20110648

[52] Kishi N, Macklis JD. MeCP2 functions largely cell-autonomously, but also non-cell-autonomously, in neuronal maturation and dendritic arborization of cortical pyramidal neurons. Exp Neurol. 2010;222(1):51–8. 20025874 10.1016/j.expneurol.2009.12.007 PMC2846301

[53] Lam PY, Peterson RT. Developing zebrafish disease models for in vivo small molecule screens. Curr Opin Chem Biol. 2019;50:37–44. 30928773 10.1016/j.cbpa.2019.02.005 PMC6800242

[54] Lee HB, Shams S, Dang Thi VH, Boyum GE, Modhurima R, Hall EM, Green IK, Cervantes EM, Miguez FE, Clark KJ. Key HPI axis receptors facilitate light adaptive behavior in larval zebrafish. Sci Rep. 2024;14(1):7759. 38565594 10.1038/s41598-024-57707-6 PMC10987622

[55] Buske C, Gerlai R. Shoaling develops with age in Zebrafish (Danio rerio). Prog Neuropsychopharmacol Biol Psychiatry. 2011;35(6):1409–15. 20837077 10.1016/j.pnpbp.2010.09.003 PMC3021101

[56] Buske C, Gerlai R. Maturation of shoaling behavior is accompanied by changes in the dopaminergic and serotoninergic systems in zebrafish. Dev Psychobiol. 2012;54(1):28–35. 21656763 10.1002/dev.20571 PMC3170669

[57] Dreosti E, Lopes G, Kampff AR, Wilson SW. Development of social behavior in young zebrafish. Frontiers in neural circuits. 2015;9:39. 26347614 10.3389/fncir.2015.00039 PMC4539524

[58] Mahabir S, Chatterjee D, Buske C, Gerlai R. Maturation of shoaling in two zebrafish strains: a behavioral and neurochemical analysis. Behav Brain Res. 2013;247:1–8. 23518435 10.1016/j.bbr.2013.03.013 PMC3646909

[59] Pham M, Raymond J, Hester J, Kyzar E, Gaikwad S, Bruce I, Fryar C, Chanin S, Enriquez J, Bagawandoss S, et al. Assessing social behavior phenotypes in adult zebrafish: shoaling, social preference, and mirror biting tests. In: Kalueff AV, Stewart AM, editors., et al., Zebrafish protocols for neurobehavioral research. Totowa: Humana Press; 2012. p. 231–46.

[60] Shams S, Khan A, Gerlai R. Early social deprivation does not affect cortisol response to acute and chronic stress in zebrafish. Stress. 2021;2021:1–9. 10.1080/10253890.2020.1807511 32781882

[61] Cachat J, Canavello PR, Elkhayat S, Bartels B, Hart PC, Elegante MF, Beeson E, Laffoon AL, Haymore WAM, Tien D, et al. Video-Aided Analysis of Zebrafish Locomotion and Anxiety-Related Behavioral Responses. Totowa: Humana Press; 2011.

[62] Shams S, Seguin D, Facciol A, Chatterjee D, Gerlai R. Effect of social isolation on anxiety-related behaviors, cortisol, and monoamines in adult zebrafish. Behav Neurosci. 2017;131(6):492–504. 29189020 10.1037/bne0000220

[63] Coverdale LE, Martyniuk CJ, Trudeau VL, Martin CC. Differential expression of the methyl-cytosine binding protein 2 gene in embryonic and adult brain of zebrafish. Brain Res Dev Brain Res. 2004;153(2):281–7. 15527897 10.1016/j.devbrainres.2004.08.009

[64] Pietri T, Roman AC, Guyon N, Romano SA, Washbourne P, Moens CB, de Polavieja GG, Sumbre G. The first mecp2-null zebrafish model shows altered motor behaviors. Front Neural Circ. 2013;7:118. 10.3389/fncir.2013.00118 PMC3712905 23874272

[65] Diotel N, Mérot Y, Coumailleau P, Gueguen MM, Sérandour AA, Salbert G, Kah O. 5-hydroxymethylcytosine marks postmitotic neural cells in the adult and developing vertebrate central nervous system. J Comp Neurol. 2017;525(3):478–97. 27414756 10.1002/cne.24077

[66] Cortelazzo A, Pietri T, De Felice C, Leoncini S, Guerranti R, Signorini C, Timperio AM, Zolla L, Ciccoli L, Hayek J. Proteomic analysis of the Rett syndrome experimental model mecp2(Q63X) mutant zebrafish. J Proteomics. 2017;154:128–33. 28062374 10.1016/j.jprot.2016.12.010

[67] Santistevan NJ, Ford CT, Gilsdorf CS, Grinblat Y. Behavioral and transcriptomic analyses of mecp2 function in zebrafish. Am J Med Genet Part B Neuropsychiatr Genet. 2024;2024:e32981. 10.1002/ajmg.b.32981 38551133

[68] Hendrich B, Tweedie S. The methyl-CpG binding domain and the evolving role of DNA methylation in animals. Trends in genetics : TIG. 2003;19(5):269–77. 12711219 10.1016/S0168-9525(03)00080-5

[69] Pappalardo-Carter DL, Balaraman S, Sathyan P, Carter ES, Chen WJ, Miranda RC. Suppression and epigenetic regulation of MiR-9 contributes to ethanol teratology: evidence from zebrafish and murine fetal neural stem cell models. Alcohol Clin Exp Res. 2013;37(10):1657–67. 23800254 10.1111/acer.12139 PMC3785568

[70] Yang H, Zhou Y, Gu J, Xie S, Xu Y, Zhu G, Wang L, Huang J, Ma H, Yao J. Deep mRNA sequencing analysis to capture the transcriptome landscape of zebrafish embryos and larvae. PLoS ONE. 2013;8(5): e64058. 23700457 10.1371/journal.pone.0064058 PMC3659048

[71] Huang HT, Kathrein KL, Barton A, Gitlin Z, Huang YH, Ward TP, Hofmann O, Dibiase A, Song A, Tyekucheva S, et al. A network of epigenetic regulators guides developmental haematopoiesis in vivo. Nat Cell Biol. 2013;15(12):1516–25. 24240475 10.1038/ncb2870 PMC3959952

[72] Gao H, Bu Y, Wu Q, Wang X, Chang N, Lei L, Chen S, Liu D, Zhu X, Hu K, et al. Mecp2 regulates neural cell differentiation by suppressing the Id1 to Her2 axis in zebrafish. J Cell Sci. 2015;128(12):2340–50. 25948585 10.1242/jcs.167874

[73] Leong WY, Lim ZH, Korzh V, Pietri T, Goh EL. Methyl-CpG binding protein 2 (Mecp2) regulates sensory function through sema5b and robo2. Front Cell Neurosci. 2015;9:481. 26733807 10.3389/fncel.2015.00481 PMC4685056

[74] Laing LV, Viana J, Dempster EL, Trznadel M, Trunkfield LA, Uren Webster TM, van Aerle R, Paull GC, Wilson RJ, Mill J, et al. Bisphenol A causes reproductive toxicity, decreases dnmt1 transcription, and reduces global DNA methylation in breeding zebrafish (Danio rerio). Epigenetics. 2016;11(7):526–38. 27120497 10.1080/15592294.2016.1182272 PMC4939919

[75] Wang Y, Zhong H, Wang C, Gao D, Zhou Y, Zuo Z. Maternal exposure to the water soluble fraction of crude oil, lead and their mixture induces autism-like behavioral deficits in zebrafish (Danio rerio) larvae. Ecotoxicol Environ Safety. 2016;1341:23–30. 10.1016/j.ecoenv.2016.08.009 27573365

[76] Jimenez-Gonzalez A, García-Concejo A, López-Benito S, Gonzalez-Nunez V, Arévalo JC, Rodriguez RE. Role of morphine, miR-212/132 and mu opioid receptor in the regulation of Bdnf in zebrafish embryos. Biochem Biophys Acta. 2016;1860(6):1308–16. 26947007 10.1016/j.bbagen.2016.03.001

[77] Garcia-Concejo A, Jimenez-Gonzalez A, Rodríguez RE. μ opioid receptor expression after morphine administration is regulated by miR-212/132 cluster. PLoS ONE. 2016;11(7): e0157806. 27380026 10.1371/journal.pone.0157806 PMC4933400

[78] Nozawa K, Lin Y, Kubodera R, Shimizu Y, Tanaka H, Ohshima T. Zebrafish Mecp2 is required for proper axonal elongation of motor neurons and synapse formation. Dev Neurobiol. 2017;77(9):1101–13. 28371371 10.1002/dneu.22498

[79] van der Vaart M, Svoboda O, Weijts BG, Espín-Palazón R, Sapp V, Pietri T, Bagnat M, Muotri AR, Traver D. Mecp2 regulates tnfa during zebrafish embryonic development and acute inflammation. Dis Model Mech. 2017;10(12):1439–51. 28993314 10.1242/dmm.026922 PMC5769600

[80] Laing LV, Viana J, Dempster EL, Uren Webster TM, van Aerle R, Mill J, Santos EM. Sex-specific transcription and DNA methylation profiles of reproductive and epigenetic associated genes in the gonads and livers of breeding zebrafish. Comp Biochem Physiol A. 2018;222:16–25. 10.1016/j.cbpa.2018.04.004 29655816

[81] Sakai C, Ijaz S, Hoffman EJ. Zebrafish models of neurodevelopmental disorders: past, present, and future. Front Mol Neurosci. 2018;11:294. 30210288 10.3389/fnmol.2018.00294 PMC6123572

[82] Pisera-Fuster A, Faillace MP, Bernabeu R. Pre-exposure to nicotine with nocturnal abstinence induces epigenetic changes that potentiate nicotine preference. Mol Neurobiol. 2020;57(4):1828–46. 31848934 10.1007/s12035-019-01843-y

[83] Ross SE, Hesselson D, Bogdanovic O. Developmental accumulation of gene body and transposon non-cpg methylation in the zebrafish brain. Front Cell Dev Biol. 2021;9: 643603. 33748137 10.3389/fcell.2021.643603 PMC7978034

[84] Baronio D, Chen YC, Panula P. Abnormal brain development of monoamine oxidase mutant zebrafish and impaired social interaction of heterozygous fish. Dis Models Mech. 2022;15(3):dmm049133. 10.1242/dmm.049133 PMC8891935 34881779

[85] Gonçalves C, Kareklas K, Teles MC, Varela SAM, Costa J, Leite RB, Paixão T, Oliveira RF. Phenotypic architecture of sociality and its associated genetic polymorphisms in zebrafish. Genes Brain Behav. 2022;21(5):e12809. 35524578 10.1111/gbb.12809 PMC9744564

[86] Gabellini C, Pucci C, De Cesari C, Martini D, Di Lauro C, Digregorio M, Norton W, Zippo A, Sessa A, Broccoli V, et al. CRISPR/Cas9-induced inactivation of the autism-risk gene setd5 leads to social impairments in zebrafish. Int J Mol Sci. 2022;24(1):167. 36613611 10.3390/ijms24010167 PMC9820161

[87] Varela T, Varela D, Martins G, Conceição N, Cancela ML. Cdkl5 mutant zebrafish shows skeletal and neuronal alterations mimicking human CDKL5 deficiency disorder. Sci Rep. 2022;12(1):9325. 35665761 10.1038/s41598-022-13364-1 PMC9167277

[88] Adrião A, Mariano S, Mariano J, Gavaia PJ, Cancela ML, Vitorino M, Conceição N. mef2ca and mef2cb double mutant zebrafish show altered craniofacial phenotype and motor behaviour. Biomolecules. 2023;13(5):805. 37238675 10.3390/biom13050805 PMC10216501

[89] Pramanik S, Bala A, Pradhan A. Zebrafish in understanding molecular pathophysiology, disease modeling, and developing effective treatments for Rett syndrome. J Gene Med. 2024;26(2): e3677. 38380785 10.1002/jgm.3677

[90] Privat M, Hansen ECA, Pietri T, Marachlian E, Uribe-Arias A, Duchemin A, Candat V, Nourin S, Sumbre G. Attractor-like circuits improve visual decoding and behavior in zebrafish. bioRxiv 2024:2024.2002.2003.578596.

[91] Landin J, Hovey D, Xu B, Lagman D, Zettergren A, Larhammar D, Kettunen P, Westberg L. Oxytocin receptors regulate social preference in zebrafish. Sci Rep. 2020;10(1):5435. 32214126 10.1038/s41598-020-61073-4 PMC7096398

[92] Baraban SC, Taylor MR, Castro PA, Baier H. Pentylenetetrazole induced changes in zebrafish behavior, neural activity and c-fos expression. Neuroscience. 2005;131(3):759–68. 15730879 10.1016/j.neuroscience.2004.11.031

[93] Canavello PR, Cachat JM, Beeson EC, Laffoon AL, Grimes C, Haymore WAM, Elegante MF, Bartels BK, Hart PC, Elkhayat SI, et al. Measuring endocrine (cortisol) responses of zebrafish to stress. In: Kalueff AV, Cachat JM, editors., et al., Zebrafish neurobehavioral protocols. Totowa: Humana Press; 2011. p. 135–42.

[94] Baraban SC, Dinday MT, Hortopan GA. Drug screening in Scn1a zebrafish mutant identifies clemizole as a potential Dravet syndrome treatment. Nat Commun. 2013;4:2410. 24002024 10.1038/ncomms3410 PMC3891590

[95] D’Amora M, Galgani A, Marchese M, Tantussi F, Faraguna U, De Angelis F, Giorgi FS. Zebrafish as an innovative tool for epilepsy modeling: state of the art and potential future directions. Int J Mol Sci. 2023;24(9):725. 10.3390/ijms24097702 PMC10177843 37175408

[96] Hoffman EJ, Turner KJ, Fernandez JM, Cifuentes D, Ghosh M, Ijaz S, Jain RA, Kubo F, Bill BR, Baier H, et al. Estrogens suppress a behavioral phenotype in zebrafish mutants of the autism risk gene, CNTNAP2. Neuron. 2016;89(4):725–33. 26833134 10.1016/j.neuron.2015.12.039 PMC4766582

[97] Baraban SC. A zebrafish-centric approach to antiepileptic drug development. Dis models Mech. 2021;14(7):dmm049080. 10.1242/dmm.049080 PMC8277967 34231838

[98] Geng Y, Peterson RT. The zebrafish subcortical social brain as a model for studying social behavior disorders. Dis Models Mech. 2019;12(8):dmm039446. 10.1242/dmm.039446 PMC6737945 31413047

[99] Kareklas K, Teles MC, Dreosti E, Oliveira RF. Autism-associated gene shank3 is necessary for social contagion in zebrafish. Mol Autism. 2023;14(1):23. 37391856 10.1186/s13229-023-00555-4 PMC10311831

[100] Weinschutz Mendes H, Neelakantan U, Liu Y, Fitzpatrick SE, Chen T, Wu W, Pruitt A, Jin DS, Jamadagni P, Carlson M, et al. High-throughput functional analysis of autism genes in zebrafish identifies convergence in dopaminergic and neuroimmune pathways. Cell Rep. 2023;42(3): 112243. 36933215 10.1016/j.celrep.2023.112243 PMC10277173

[101] Zhou H, Wu W, Zhang Y, He H, Yuan Z, Zhu Z, Zhao Z. Selective preservation of cholinergic MeCP2 rescues specific Rett-syndrome-like phenotypes in MeCP2(stop) mice. Behav Brain Res. 2017;322(Pt A):51–9. 28093257 10.1016/j.bbr.2017.01.023

[102] Bhave SA, Uht RM. CpG methylation and the methyl CpG binding protein 2 (MeCP2) are required for restraining corticotropin releasing hormone (CRH) gene expression. Mol Cell Endocrinol. 2017;454:158–64. 28655627 10.1016/j.mce.2017.06.024

[103] Heck AL, Thompson MK, Uht RM, Handa RJ. Sex-dependent mechanisms of glucocorticoid regulation of the mouse hypothalamic corticotropin-releasing hormone gene. Endocrinology. 2020;161:1. 31754709 10.1210/endocr/bqz012 PMC7188085

[104] McGill BE, Bundle SF, Yaylaoglu MB, Carson JP, Thaller C, Zoghbi HY. Enhanced anxiety and stress-induced corticosterone release are associated with increased Crh expression in a mouse model of Rett syndrome. Proc Natl Acad Sci USA. 2006;103(48):18267–72. 17108082 10.1073/pnas.0608702103 PMC1636379

[105] McEwen BS, Gray JD, Nasca C. 60 years of neuroendocrinology: redefining neuroendocrinology: stress, sex and cognitive and emotional regulation. J Endocrinol. 2015;226(2):T67-83. 25934706 10.1530/JOE-15-0121 PMC4515381

[106] Shahbazian M, Young J, Yuva-Paylor L, Spencer C, Antalffy B, Noebels J, Armstrong D, Paylor R, Zoghbi H. Mice with truncated MeCP2 recapitulate many Rett syndrome features and display hyperacetylation of histone H3. Neuron. 2002;35(2):243–54. 12160743 10.1016/s0896-6273(02)00768-7

[107] Stearns NA, Schaevitz LR, Bowling H, Nag N, Berger UV, Berger-Sweeney J. Behavioral and anatomical abnormalities in Mecp2 mutant mice: a model for Rett syndrome. Neuroscience. 2007;146(3):907–21. 17383101 10.1016/j.neuroscience.2007.02.009

[108] Wither RG, Colic S, Bardakjian BL, Snead OC 3rd, Zhang L, Eubanks JH. Electrographic and pharmacological characterization of a progressive epilepsy phenotype in female MeCP2-deficient mice. Epilepsy Res. 2018;140:177–83. 29414525 10.1016/j.eplepsyres.2018.01.015

[109] Zhang W, Peterson M, Beyer B, Frankel WN, Zhang ZW. Loss of MeCP2 from forebrain excitatory neurons leads to cortical hyperexcitation and seizures. J Neurosci. 2014;34(7):2754–63. 24523563 10.1523/JNEUROSCI.4900-12.2014 PMC3921436

[110] Shahbazian MD, Antalffy B, Armstrong DL, Zoghbi HY. Insight into Rett syndrome: MeCP2 levels display tissue- and cell-specific differences and correlate with neuronal maturation. Hum Mol Genet. 2002;11(2):115–24. 11809720 10.1093/hmg/11.2.115

